# Assessment of cochlear toxicity in response to chronic 3,3′-iminodipropionitrile in mice reveals early and reversible functional loss that precedes overt histopathology

**DOI:** 10.1007/s00204-020-02962-5

**Published:** 2021-01-25

**Authors:** Erin A. Greguske, Jordi Llorens, Sonja J. Pyott

**Affiliations:** 1 Departament de Ciències Fisiològiques and Institut de Neurociències, Universitat de Barcelona, 08907 L’Hospitalet de Llobregat, Catalonia Spain; 2 Institut d’Investigació Biomèdica de Bellvitge (IDIBELL), 08907 L’Hospitalet de Llobregat, Catalonia Spain; 3 Department of Otorhinolaryngology/Head and Neck Surgery, University Medical Center Groningen, University of Groningen, 9700 RB Groningen, The Netherlands

**Keywords:** Cochlea, Vestibule, Hair cell, Afferent synapse, Ototoxicity, Acquired hearing loss, Cochlear synaptopathy

## Abstract

The peripheral auditory and vestibular systems rely on sensorineural structures that are vulnerable to ototoxic agents that cause hearing loss and/or equilibrium deficits. Although attention has focused on hair cell loss as the primary pathology underlying ototoxicity, evidence from the peripheral vestibular system indicates that hair cell loss during chronic exposure is preceded by synaptic uncoupling from the neurons and is potentially reversible. To determine if synaptic pathology also occurs in the peripheral auditory system, we examined the extent, time course, and reversibility of functional and morphological alterations in cochleae from mice exposed to 3,3′-iminodipropionitrile (IDPN) in drinking water for 2, 4 or 6 weeks. Functionally, IDPN exposure caused progressive high- to low-frequency hearing loss assessed by measurement of auditory brainstem response wave I absolute thresholds and amplitudes. The extent of hearing loss scaled with the magnitude of vestibular dysfunction assessed behaviorally. Morphologically, IDPN exposure caused progressive loss of outer hair cells (OHCs) and synapses between the inner hair cells (IHCs) and primary auditory neurons. In contrast, IHCs were spared from ototoxic damage. Importantly, hearing loss consistent with cochlear synaptopathy preceded loss of OHCs and synapses and, moreover, recovered if IDPN exposure was stopped before morphological pathology occurred. Our observations suggest that synaptic uncoupling, perhaps as an early phase of cochlear synaptopathy, also occurs in the peripheral auditory system in response to IDPN exposure. These findings identify novel mechanisms that contribute to the earliest stages of hearing loss in response to ototoxic agents and possibly other forms of acquired hearing loss.

**Supplementary Information:**

The online version contains supplementary material available at 10.1007/s00204-020-02962-5.

## Introduction

The auditory and vestibular systems in the inner ear rely on sensorineural structures that share many structural, molecular, and physiological features, including sensitivity to a variety of ototoxic agents that cause hearing loss and/or equilibrium deficits (Schacht et al. 2012). Aminoglycoside-induced auditory and vestibular toxicity have been demonstrated in both humans and animal models (Forge and Schacht 2000; Jiang et al. 2017; Van Hecke et al. 2017). In humans, the antineoplastic drug cisplatin is well known to cause hearing loss. In both humans and animal models, studies indicate that cisplatin is also toxic to the vestibular system (Callejo et al. 2017; Kitsigianis et al. 1988; Takimoto et al. 2016). Experimentally, several low molecular weight nitriles have also been demonstrated to be ototoxic (Crofton et al. 1994; Gagnaire et al. 2001; Llorens et al. 1993; Saldana-Ruiz et al. 2012; Soler-Martin et al. 2007).

In both the peripheral auditory and vestibular systems, ototoxic loss of the sensory hair cells (HCs) responsible for mechanotransduction has been particularly well documented. The vulnerability of HCs to ototoxicity varies depending on their subtype and also location within the sensory epithelia (Crofton et al. 1994; Forge and Schacht 2000; Llorens et al. 1993; Schacht et al. 2012; Soler-Martin et al. 2007). For example, in the cochlea, outer HCs (OHCs) degenerate earlier and/or with lower doses than inner HCs (IHCs), and, in the vestibule, type I HCs are more susceptible than type II HCs. Regionally, in the cochlea, the basal turns, which encode higher frequency sounds, are more vulnerable than the apical turns, which encode lower frequency sounds. Of the five sensory epithelia in the vestibule, the utricular macula and the three cristae in the semi-circular canals are more vulnerable than the saccular macula.

Although attention has historically focused on HC degeneration as the primary pathology underlying ototoxicity, other pathologies have been recently identified. Evidence from both rats (Sedó-Cabezón et al. 2015) and mice (Greguske et al. 2019) exposed to 3,3′-iminodipropionitrile (IDPN) in drinking water indicates that damage to the specialized synaptic contacts between the vestibular type I HCs and the calyx-shaped afferent endings of the vestibular neurons is an early event in chronic vestibular toxicity. Specifically, in these studies, ototoxicity triggered the loss of adhesion proteins between the type I HCs and calyx afferent terminals as well as the loss of both presynaptic ribbons—the specialized structures for neurotransmitter release from the HCs—and postsynaptic densities and postsynaptic glutamate receptor clusters found on the calyx terminals. In both animal models, these pathological alterations were reversible if exposure was terminated promptly. Importantly, significant vestibular dysfunction was associated with damage to the synaptic contacts and recovered following cessation of IDPN exposure, presumably due to synaptic recovery. Previous work in rats had shown that extended chronic IDPN exposure led to extrusion of the HCs (Seoane et al. 2001). Together, these findings suggest that synaptic uncoupling of the HCs from the afferent calyx terminals is the initial step in chronic ototoxic stress that precedes, and may trigger, extrusion, and finally loss of HCs.

Although evidence is more limited, a few studies suggest either directly or indirectly that synaptic uncoupling also plays a significant and primary role in ototoxicity in the auditory system. First, post-mortem examination of patients who received either short- (Hinojosa and Lerner 1987) or long (Sone et al. 1998)-term treatment with aminoglycosides have identified temporal bones with either no or only scattered loss of HCs, but notable degeneration of the primary auditory neurons—the type I spiral ganglion neurons (SGNs). Second, researchers examining mice exposed acutely to the aminoglycoside antibiotic gentamicin (in combination with the diuretic furosemide to potentiate aminoglycoside ototoxicity) found that the loss of the auditory neuron afferent dendrites contacting the IHCs preceded and also exceeded loss of the HCs; they hypothesized that gentamicin-induced damage to cochlear innervation occurs independently of HC loss (Ruan et al. 2014). Third, substantial evidence demonstrates that the earliest and perhaps most common mechanism of hearing impairment in response to noise exposure (Kujawa and Liberman 2009) and aging (Sergeyenko et al. 2013) results not from loss of HCs but rather from cochlear synaptopathy, the loss of the synapses between the auditory neuron afferent dendrites and the IHCs (Liberman and Kujawa 2017). Shared mechanisms underlie the triad of age-related, noise-induced, and ototoxic hearing loss (Yang et al. 2015) and excitotoxicity, in particular, may contribute to cochlear synaptopathy associated with ototoxicity (Basile et al. 1996; Duan et al. 2000). Thus, similar to the vestibular system, synaptic uncoupling may be the earliest and perhaps reversible pathology associated with ototoxicity in the auditory system.

In this study, we examined the time course of functional and morphological alterations in the peripheral auditory system in response to chronic ototoxic exposure. We were particularly interested if and to what extent synaptic uncoupling and/or cochlear synaptopathy together with HC loss contributed to hearing loss and, moreover, if hearing loss was reversible following cessation of ototoxic exposure. To this end, we examined mice exposed chronically to IDPN in drinking water. IDPN is the best characterized ototoxic nitrile and induces both vestibular and auditory toxicity reliably and dose-dependently in a variety of species (Crofton et al. 1994; Llorens et al. 1993; Llorens and Rodriguez-Farre 1997; Seoane et al. 2001; Soler-Martin et al. 2007). We found that several pathological events, not only HC loss, are associated with hearing loss following IDPN exposure and that hearing loss recovered following limited exposure. These findings provide insight into the earliest and most reversible stages of ototoxicity and are likely shared by other forms of acquired hearing loss.

## Materials and methods

### Animals

All experiments were approved by the animal ethics committee of the University of Groningen (UG) and University Medical Center Groningen (UMCG) and complied with guidelines for animal experiments from the UG/UMCG, the Netherlands, and European animal welfare law. Male 129S2/SvPasCrl mice (5 weeks of age) were obtained from Charles River (Germany) and housed in the UMCG central animal facility. Research examining the sex- and species-dependent effects of IDPN exposure observed sufficient vestibular toxicity without excessive systemic toxicity in male 129S1/SvImJ mice (Greguske et al. 2019). Therefore, only male mice were utilized in these experiments. Mice were housed in 12:12 h light:dark cycle and allowed ad libitum access to food and water. Mice were monitored daily and weighed weekly (Supplemental Table 1 and Supplemental Fig. 1). A total of 83 mice were used in these experiments. As described in more detail below, all mice underwent baseline assessment and then were divided into either a control group or an exposure group. Mice in both groups were re-assessed at 2, 4, and 6 weeks. A subset of mice undergoing exposure for 2 weeks was allowed to recover for an additional 2 and 4 weeks (recovery/washout). The number of animals in each of the experimental groups are included in the text, figures, and tables.

## Ototoxic exposure

IDPN exposure was based on a previously established protocol (Greguske et al. 2019) with minor adjustments to the exposure timeline. Briefly, mice were allowed 1 week to acclimatize after arrival. Mice were then randomly assigned to either the control or treatment groups. Mice selected for treatment were exposed to 30 mM IDPN in their drinking water (*n* = 44); control mice were given standard drinking water (*n* = 39). Water bottles were changed weekly and weighed to estimate the dose received.

## Vestibular dysfunction rating

To assess peripheral vestibular function in IDPN-treated, control, and washout mice, three behaviors during an open field observation and three anti-gravity reflexes were scored as described previously in mice (Boadas-Vaello et al. 2007, 2009; Greguske et al. 2019; Saldana-Ruiz et al. 2012, 2013; Soler-Martin et al. 2007) and rats (Boadas-Vaello et al. 2005; Llorens et al. 1993; Llorens and Rodriguez-Farre 1997; Sedó-Cabezón et al. 2015). During the open field observation, mice were placed in an empty rat cage and observed for 1 min in silence for head bobbing (intermittent extreme backward extension of the neck), circling (stereotyped circulatory ambulation), and retropulsion (backward movement). For the anti-gravity reflexes, mice were assessed using the tail-lift reflex, the air-righting reflex, and the contact-inhibition of the righting reflex. Scores for both types of tests were scaled from 0 (normal function) to 4 (highly dysfunctional); therefore, the total vestibular dysfunction rating (VDR) for the entire test battery ranged from 0 to 24.

## Auditory brainstem response measurements

Mice were anesthetized with 75 mg/kg ketamine and 1 mg/kg dexmedetomidine via subcutaneous injection into the loose skin between the shoulders and neck. After confirmation of anesthesia, auditory brainstem responses (ABRs) were measured as described previously (Reijntjes et al. 2019). Both click and pure tone (8, 16, 24, 32 kHz) ABRs were recorded from the right ear only to reduce the total time under anesthesia. Dedicated hardware and software were used to generate acoustic stimuli (Intelligent Hearing Systems). ABRs were recorded with an initial intensity of 20 dB SPL and then increased to 90 dB SPL in 5 dB SPL increments. Electrode recordings were amplified 100X, bandpass filtered between 30 and 3000 Hz, and averaged over 512 recordings. ABR wave I absolute thresholds were set at the first intensity where the peak of wave I was distinguishable above the noise and appeared consistently in subsequent recordings. ABR wave I amplitudes were set by marking the peak of wave I and its preceding trough. ABR wave I latencies were calculated as the time delay for which the peak of wave I appeared after the click stimulus or tone burst was presented.

## Isolation and immunofluorescence staining of the auditory and vestibular epithelia

For collection of auditory epithelia (organs of Corti) and vestibular epithelia, mice were euthanized by decapitation immediately following the final ABR measurements and while still under anesthesia. The bony labyrinths, which house the inner ear sensory epithelia, of both ears were wholly extracted from the temporal bones and prepared for immunofluorescence as described previously (McLean et al. 2009; Schuth et al. 2014). Small holes were chiseled into the bone above the utricle and in the apical end of the cochlea to allow penetration of ice-cold fixation solution, 4% paraformaldehyde (PFA) in phosphate buffered saline (PBS), for 1 h. After fixation, the bony labyrinths were then placed into ice-cold PBS, and the cochlear and vestibular sensory epithelia were carefully extracted. Tissue was stored in cold PBS until further processing.

Isolated sensory epithelia were dissected from the bony labyrinth and immediately placed into blocking buffer (PBS with 5% normal goat serum, 4% Triton X-100, and 1% saponin) for at least 1 h and then incubated overnight with the primary antibodies diluted in blocking buffer. Two primary antibody combinations for the organs of Corti were used as follows: rabbit polyclonal anti-prestin (gift from Dr. Mary Ann Cheatham; 1:3000), mouse monoclonal (IgG1) anti-CTBP2/ribeye (BD Biosciences, 612,044; 1:500), and mouse monoclonal (IgG2a) anti-GluA2 (Millipore, MAP397; 1:300) or rabbit polyclonal anti-calretinin (Millipore, AB1550; 1/500) and mouse monoclonal (IgG1) anti-CASPR1 (NeuroMab, K65/35 or 75-001; 1:300. Primary antibody combinations for the vestibular sensory epithelia were as follows: rabbit polyclonal anti-MYO7A (Prestige Antibodies, HPA028918; 1:500), anti-CTBP2/ribeye (IgG1, 1:500), and anti-GluA2 (IgG2a, 1:300) or anti-MYO7A (rabbit, 1:500), and anti-CASPR1 (IgG1, 1:300). Samples were rinsed 3 times 10 min with PBS with 0.6% Triton-X 100 (PBT) and then incubated with the secondary antibodies for at least 4 h. The same secondary antibodies were used for all primary antibody combinations for both the organs of Corti and vestibular sensory epithelia: AlexaFluor 488 goat anti-mouse (IgG1, ThermoFisher, A-21121), AlexaFluor 568 goat anti-rabbit (IgG, ThermoFisher, A-11011), and AlexaFluor 647 goat anti-mouse (IgG2a, ThermoFisher, A-21241). All secondary antibodies used were diluted 1:500 in blocking buffer. All incubations and rinses were performed on a rocking platform at room temperature. Incubations with secondary antibodies were performed in the dark. Organs of Corti and vestibular sensory epithelia were mounted and stored at 4 °C.

## Cochlear place-frequency maps

To identify tonotopic regions in the organ of Corti, cochlear place-frequency maps were determined for each organ of Corti from each ear of each mouse. Briefly, low magnification micrographs were obtained using a Leica DM4000b microscope with a 5X objective. A montaged image of individual cochlear sections (generally 2–3 sections) was constructed for each organ of Corti using Fiji ImageJ. Tonotopic maps were overlaid using the mouse cochlear frequency map determined previously (Müller et al. 2005) in Fiji ImageJ (using a plugin found at: https://www.masseyeandear.org/research/otolaryngology/eaton-peabody-laboratories/histology-core).

## Confocal microscopy

Confocal image *z*-stacks of the organs of Corti and vestibular sensory epithelia were obtained using a Leica TCS SP8 confocal microscope. Images were taken with the 63X oil immersion objective with a resolution of 1024X1024 pixels at a speed of 400 Hz. The *z*-step was optimized to 0.3 µm for all images. *z*-stacks ranged in thickness from 6 to 13 µm (for utricle) or 25–50 µm (for organs of Corti) to encompass the entire synaptic pole of the sensory HCs. For organs of Corti, image *z*-stacks were taken at the 8, 16, 24, and 32 kHz frequencies with an optical zoom of 1. For the utricle, image *z*-stacks were taken in the striola of the utricular macula with an optical zoom of 2.

## Image analysis

The majority of quantitative image analysis was performed using Imaris v7.6. The number of cochlear IHCs was determined by CTBP2-immunolabeling of the IHC nuclei. The number of cochlear OHCs was determined by prestin-immunolabeling of the OHC lateral walls. The number of vestibular HCs was determined by MYO7A-immunolabeling in the striola (central region) of the utricular macula. To compare numbers across regions of interest (ROIs), IHC and OHC numbers were relative to a given length (100 µm) and the number of vestibular HCs were relative to a given area (100 µm^2^).

For both organs of Corti and vestibular sensory epithelia, CTBP2-labeled HC presynaptic ribbons and GluA2-labeled postsynaptic glutamate receptors were detected using the “spots” function. Colocalization of pre- and post-synaptic elements was determined using the integrated MatLab function “colocalize spots”. CTBP2-positive ribbons and GluA2-positive postsynapses were considered colocalized when they were within 1 µm of each other. All spots and colocalizations were visually confirmed. The number of synaptic elements per HC was determined by dividing the total number of synaptic elements by the number of HCs in the ROI. In the organ of Corti, at least 10 IHCs were included for each analysis. In the striola of the utricular macula, all complete HCs within the ROI were analyzed.

To quantify CASPR1 immunoreactivity in the organs of Corti, the “measure” function was used to determine the lengths of the (first) CASPR1-labeled heminodes of type I SGN afferent terminals contacting the IHCs. At least six measurements were made per IHC, and at least six IHCs were analyzed per frequency region per organ of Corti (that is, per animal). To quantify the fraction of vestibular HCs contacted by CASPR1-labeled calyces in the striola of the utricular macula, the total number of MYO7A-labeled HCs was counted and divided by the total number of CASPR1-labeled calyces in the same ROI.

## Data analysis

All data were analyzed with the GraphPad Prism 8 program and presented as mean ± SEM. unless otherwise indicated. Data were analyzed by the appropriate one-way ANOVA models with multiple comparisons or mixed effects analyses (Tukey's or Dunnett's method), or by unpaired t tests. Statistical tests are provided in the text. Statistical significance was identified when *p* values were less than 0.05. *p* values less than 0.0005 are reported as *p* ≪ 0.05.

## Results

### IDPN exposure causes progressive high to low frequency hearing loss

**Fig. 1:**
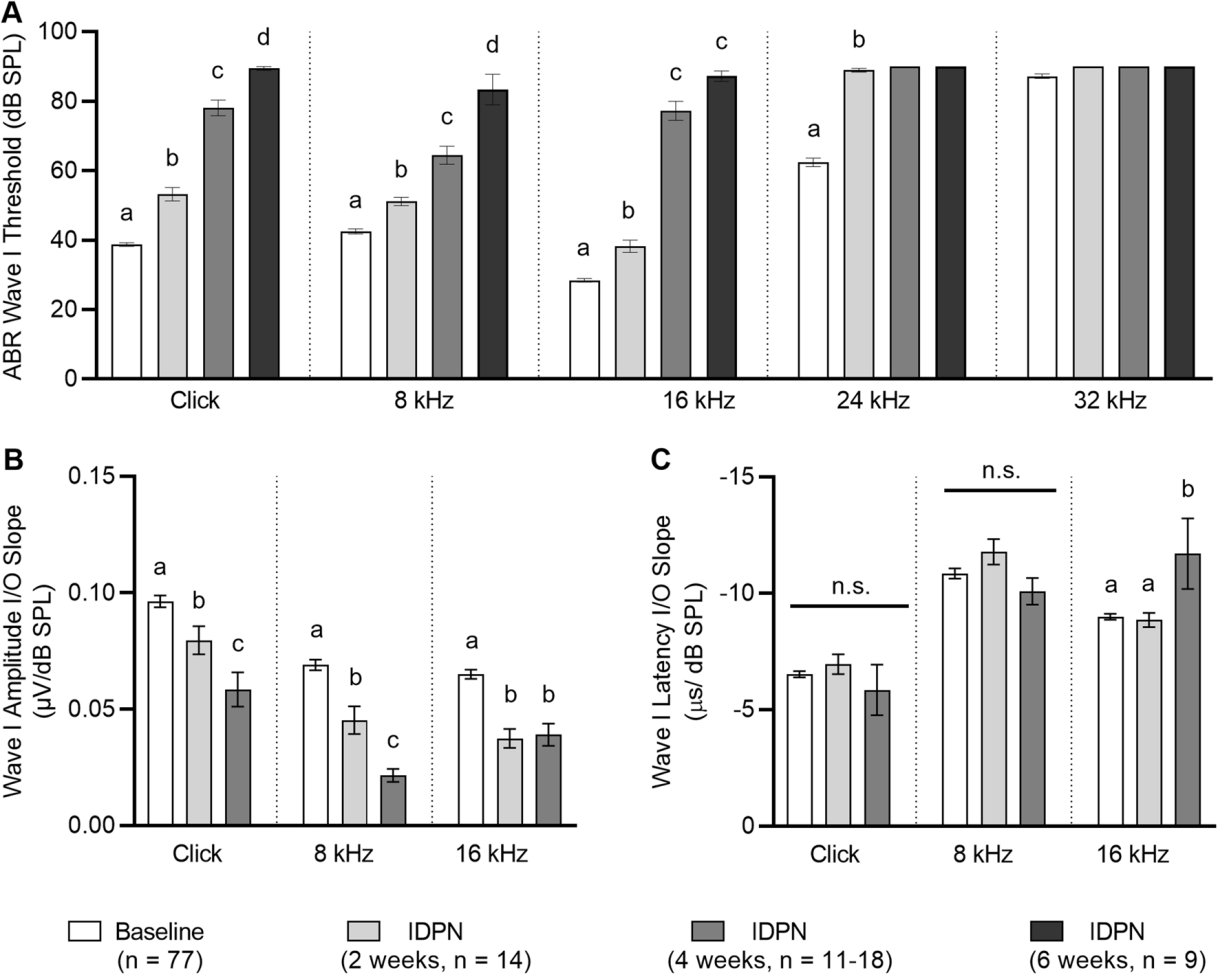
IDPN exposure causes progressive high to low frequency hearing loss assessed by ABR measurements. **a**–**c** Mean absolute ABR wave I thresholds (**a**), wave I amplitude I/O slopes (**b**), and wave I latency I/O slopes (**c**) are shown across exposure durations for click stimuli and the indicated pure tone frequencies. Wave I thresholds increase progressively from high to low frequencies with increasing IDPN exposure. Wave I amplitudes decrease progressively from high to low frequencies with increasing IDPN exposure. Wave I latencies show no significant (ns) changes across frequencies in response to IDPN exposure. In all panels, letters indicate comparisons that are significantly different. Exposure durations are indicated as baseline (white bars), 2 weeks (light grey bars), 4 weeks (dark grey bars), and 6 weeks (black bars). The numbers (*n*) of animals assessed are indicated

We assessed hearing loss in response to IDPN exposure using ABR measurements (Fig. 1). ABR wave I thresholds in response to click stimuli and pure tones (8, 16, 24, and 32 kHz) were measured at baseline (0 weeks) and again after 2, 4, and 6 weeks of exposure to IDPN (Fig. 1a; Table 1). Even at baseline, thresholds at 32 kHz were greater than 80 dB SPL, consistent with previous reports of accelerated age-related hearing loss in this strain (Zheng et al. 1999). Statistically significant increases in thresholds were observed for click stimuli and all pure tones examined as early as 2 weeks after exposure, with greater threshold shifts observed for higher frequencies (24 kHz) than lower frequencies (8 and 16 kHz). After 6 weeks of exposure, thresholds for click stimuli and all pure tones were greater than 80 dB SPL. To evaluate the effect of IDPN exposure, separate one-way ANOVA analyses were conducted for click, 8, 16, and 24 kHz data with duration of exposure (weeks) as the factor. In all cases, statistically significant differences were detected [*F*(3,114) = 388.5, *F*(3,114) = 101.2, *F*(3,114) = 453.9, *F*(3,114) = 83.9, respectively, all *p* ≪ 0.05].

**Table 1:** Wave I ABR absolute thresholds and wave I amplitude and latency I/O slopes following IDPN exposure

Measure	Treatment	Click	8 kHz	16 kHz	24 kHz	32 kHz
Wave I absolute thresholds (dB SPL)	Baseline (*n* = 77)	38.7 ± 0.5	42.5 ± 0.7	28.4 ± 0.5	62.4 ± 1.2	87.2 ± 0.7
	IDPN (2 weeks, *n* = 14)	53.2 ± 1.9	51.1 ± 1.2	38.2 ± 1.8	88.9 ± 0.6	> 90
	IDPN (4 weeks, *n* = 18)	78.1 ± 2.2	64.4 ± 2.6	77.2 ± 2.7	> 90	> 90
	IDPN (6 weeks, *n* = 9)	89.4 ± 0.6	83.3 ± 4.4	87.2 ± 1.5	> 90	> 90
Wave I amplitude I/O slopes (µV/dB SPL)	Baseline (*n* = 77)	0.096 ± 0.003	0.069 ± 0.002	0.065 ± 0.002	–	–
	IDPN (2 weeks, *n* = 14)	0.080 ± 0.006	0.045 ± 0.006	0.037 ± 0.004	–	–
	IDPN (4 weeks, *n* = 13–17)	0.058 ± 0.007 (*n* = 14)	0.022 ± 0.003 (*n* = 17)	0.039 ± 0.005 (*n* = 13)	–	–
Wave I latency I/O slopes (µs/dB SPL)	Baseline (*n* = 77)	−6.53 ± 0.14	−10.86 ± 0.22	−9.00 ± 0.13	–	–
	IDPN (2 weeks, *n* = 14)	−6.96 ± 0.43	−11.79 ± 0.55	−8.86 ± 0.30	–	–
	IDPN (4 weeks, *n* = 10–16)	−5.85 ± 1.08 (*n* = 14)	−10.09 ± 0.57 (*n* = 16)	−11.71 ± 1.52 (*n* = 10)	–	–

In addition to ABR wave I absolute thresholds, we also examined the effects of IDPN exposure on wave I amplitude and latency input/output (I/O) slopes (Fig. 1b, c; Table 1). These slopes indicate the steepness by which amplitudes increase and the latencies decrease simultaneously as a function of increasing stimulus intensities. ABR wave I absolute thresholds were greater than 80 dB SPL when measured in response to (1) 32 kHz pure tones at baseline, (2) 24 kHz pure tones after 2 weeks of IDPN exposure, and (3) click stimuli and all pure tones tested after 6 weeks of IDPN exposure. Therefore, I/O slopes were not calculated for these three conditions. For the remaining data, wave I amplitude I/O slopes showed statistically significant loss of steepness for click stimuli and 8 and 16 kHz pure tones [*F*(2,102) = 17.4, *F*(2,105) = 45.4, *F*(2,101) = 25.4, respectively, all *p* ≪ 0.05]. There is statistically significant loss of steepness for click stimuli and 8 and 16 kHz pure tones after 2 weeks of IDPN exposure (*p* = 0.037 for click and *p* ≪ 0.05 for both 8 and 16 kHz). For click stimuli and 8 kHz pure tones, steepness was further significantly reduced after an additional 2 weeks of IDPN exposure (that is, after 4 weeks of total exposure; both *p* ≪ 0.05); the slope steepness for 16 kHz pure tones remained at a reduced state after 4 weeks of IDPN exposure (*p* ≪ 0.05, Fig. 1b). Statistically significant loss of steepness was observed for the 16 kHz pure tone [*F*(2,98) = 10.4, *p* ≪ 0.05] after 4 weeks of IDPN exposure only (Fig. 1c). In contrast, wave I latency I/O functions showed no statistically significant differences after either 2 or 4 weeks of exposure to IDPN for click stimuli or the 8 kHz pure tone.

To validate that the changes in auditory function observed in response to IDPN exposure were not confounded by age-related hearing loss (Zheng et al. 1999), we also examined ABR wave I absolute thresholds as well as wave I amplitude and latency I/O functions at baseline (0 weeks) and again after 2, 4, and 6 weeks in control mice not exposed to IDPN (Supplemental Fig. 2 and Supplemental Table 2). No significant differences in any of these measures were observed, indicating that age-related hearing loss is not contributing to the progressive, high- to low-frequency hearing loss observed in response to IDPN exposure.

## IDPN exposure causes progressive loss of vestibular function that scales with hearing loss

**Fig. 2:**
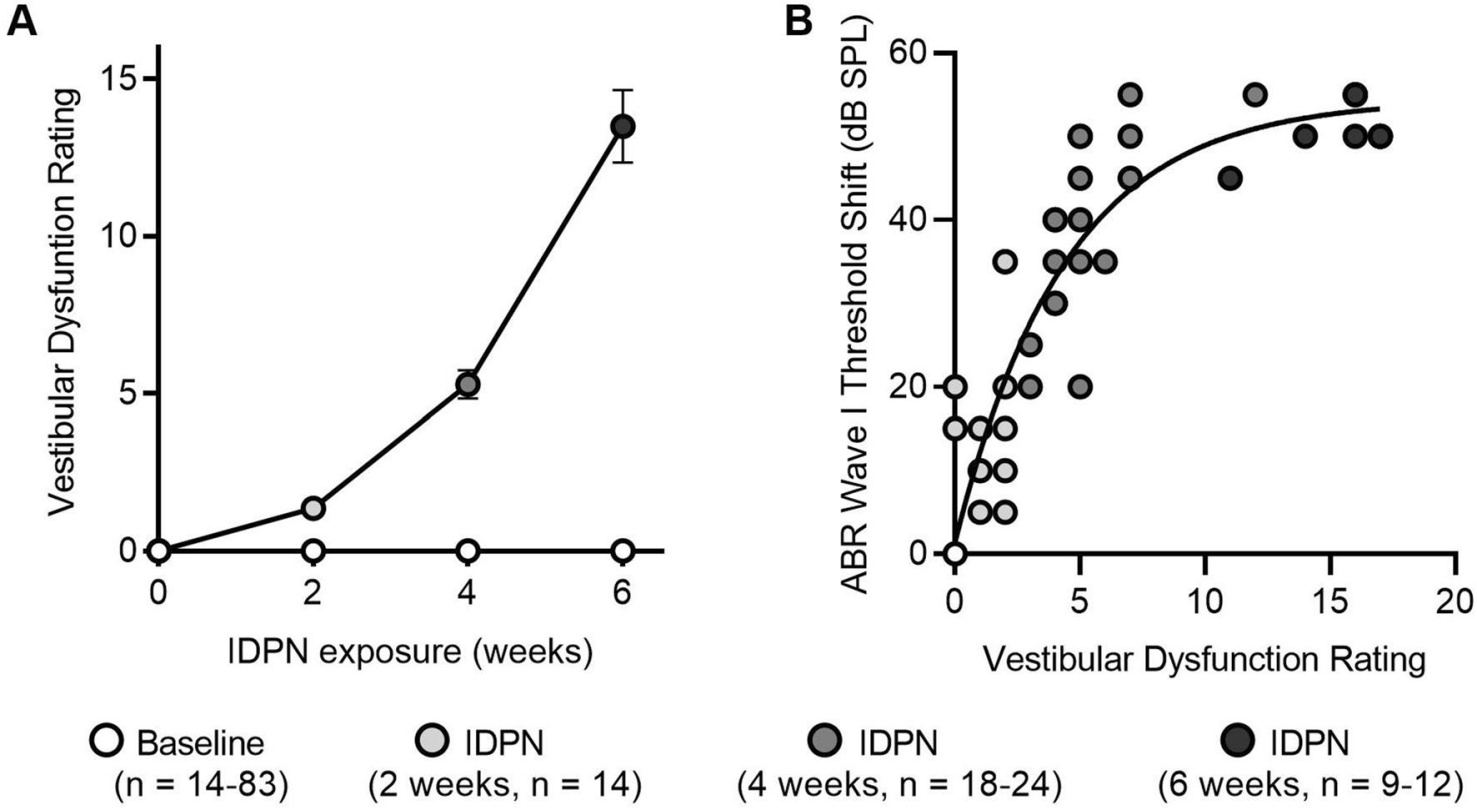
IDPN exposure causes progressive loss of vestibular function that scales with hearing loss. **a** Vestibular dysfunction (assessed by rating behavior and reflexes) is shown as a function of IDPN exposure and indicates worsening vestibular function with increasing IDPN exposure. **b** Auditory dysfunction (assessed by shifts in the ABR wave I thresholds to click stimuli) is shown as a function of vestibular dysfunction and is best fit with a single order exponential, indicating that auditory dysfunction worsened more quickly compared to vestibular dysfunction following IDPN exposure. In both panels, exposure durations include baseline (white circles), 2 weeks (light grey circles), 4 weeks (dark grey circles), and 6 weeks (black circles). The numbers (*n*) of animals assessed are indicated

To assess if the severity of hearing loss scaled with vestibular dysfunction, we quantified vestibular dysfunction (Fig. 2a and Table 2) and then examined the relationship between ABR wave I absolute threshold shifts for click stimuli and vestibular dysfunction (Fig. 2b; Table 2). As reported previously in a related strain of mice (Greguske et al. 2019) and in rats (Boadas-Vaello et al. 2005; Llorens et al. 1993; Llorens and Rodriguez-Farre 1997; Sedó-Cabezón et al. 2015), vestibular dysfunction increased with increasing IDPN exposure. Increasing vestibular dysfunction was observed in mice after 2, 4, and 6 weeks of IDPN exposure in comparison to control mice, which showed no vestibular dysfunction across the same time course. There was a positive correlation between hearing loss (ABR wave I absolute threshold shifts) and vestibular dysfunction (Fig. 2b). The relationship was nonlinear and best fit with a single order exponential, indicating that, in response to IDPN exposure, auditory dysfunction (at least as assessed by ABR wave I thresholds) worsened more quickly than vestibular dysfunction (at least as assessed using observations of behavior and reflexes).

**Table 2:** Vestibular dysfunction ratings (VDRs) and wave I ABR absolute threshold shifts following IDPN exposure

Treatment	VDR	Wave I ABR absolute threshold shift (dB SPL)
Control (Baseline, 2, 4, 6 weeks, *n* = 14–83)^a^	0 ± 0	0 ± 0
IDPN (2 weeks, *n* = 14)	1.4 ± 0.2	15 ± 2.0
IDPN (4 weeks, *n* = 18–24)	5.3 ± 0.4	38.1 ± 2.5
IDPN (6 weeks, *n* = 9–12)	13.5 ± 1.2	50.6 ± 1.0

^a^VDRs for control mice at 0 (*n* = 83), 2 (*n* = 14), 4 (*n* = 25), and 6 (*n* = 19) weeks were always equal to 0

## IDPN exposure causes loss of cochlear outer but not inner hair cells

**Fig. 3:**
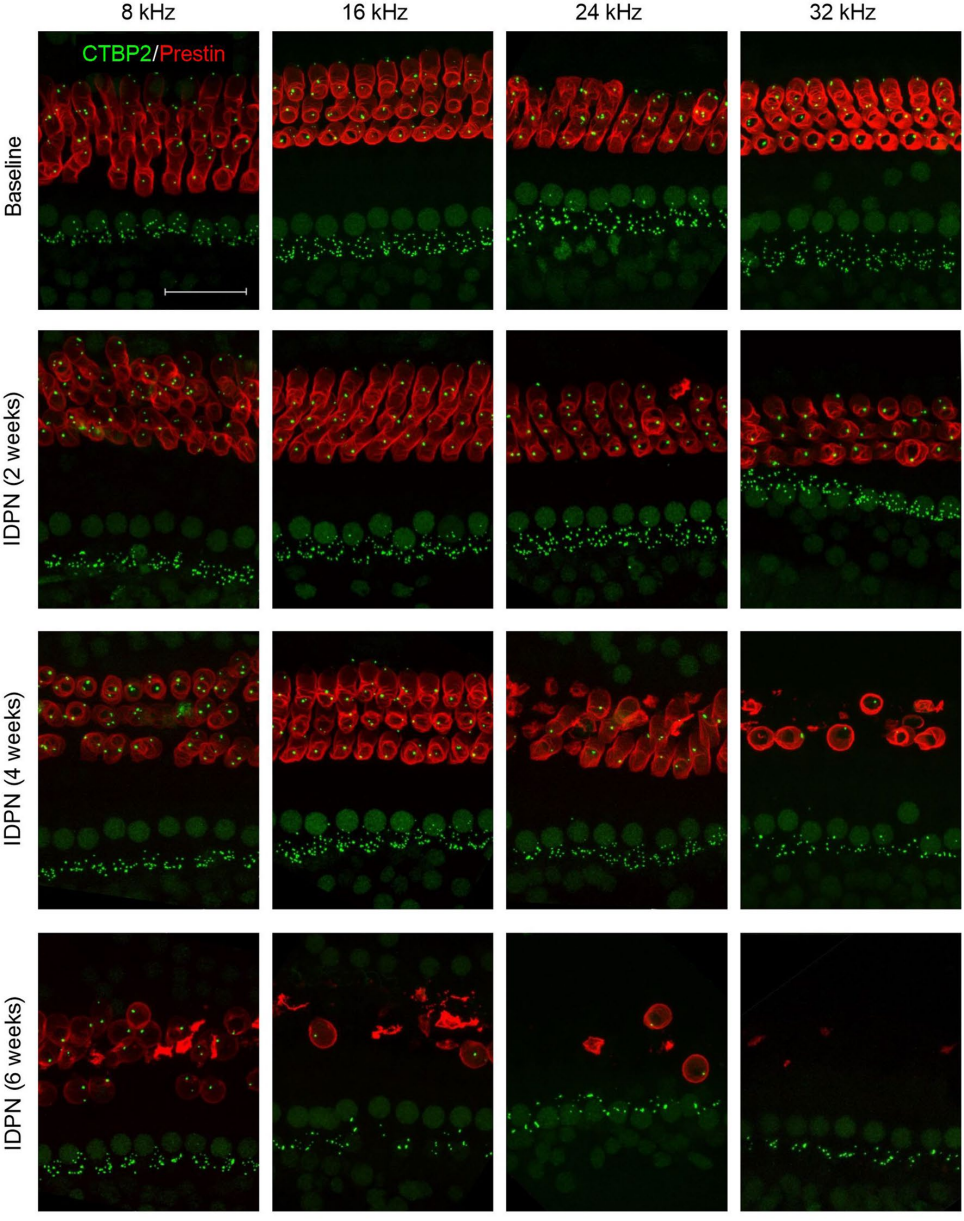
IDPN exposure causes progressive damage to the sensorineural structures of the organ of Corti. *Z*-projections through the organs of Corti immunostained with anti-CTBP2 to detect IHC nuclei and presynaptic ribbons (green) and anti-prestin (red) to detect OHCs are shown at four tonotopic regions (corresponding to 8, 16, 24, and 32 kHz) at the indicated exposure durations (baseline, 2 weeks, 4 weeks, and 6 weeks). Increasing IDPN exposure causes progressive loss of the OHCs and presynaptic ribbons from high to low frequency regions. Scale bar equals 25 µm (color figure online)

To determine the cochlear pathology underling the loss of auditory function, we examined isolated organs of Corti, from mice at baseline (0 weeks) and after 2, 4, or 6 weeks of exposure to IDPN (Fig. 3). Epithelia were immunostained with anti-prestin to detect OHCs and anti-CTBP2 to detect IHCs (more specifically, the IHC nuclei) and examined at four different frequency regions (8, 16, 24, and 32 kHz). Epithelia isolated from control mice showed the expected pattern of three rows of OHCs and a single row of IHCs, with no missing HCs in any of the frequency regions examined. After 2 weeks of IDPN exposure, OHCs and IHCs were intact. However, after 4 weeks of IDPN exposure, OHCs were missing from the higher frequency regions (24 and 32 kHz), whereas IHCs appeared intact. Finally, after 6 weeks of IDPN exposure, OHCs were either entirely absent or missing from most frequency regions, whereas IHCs remained intact.

**Fig. 4:**
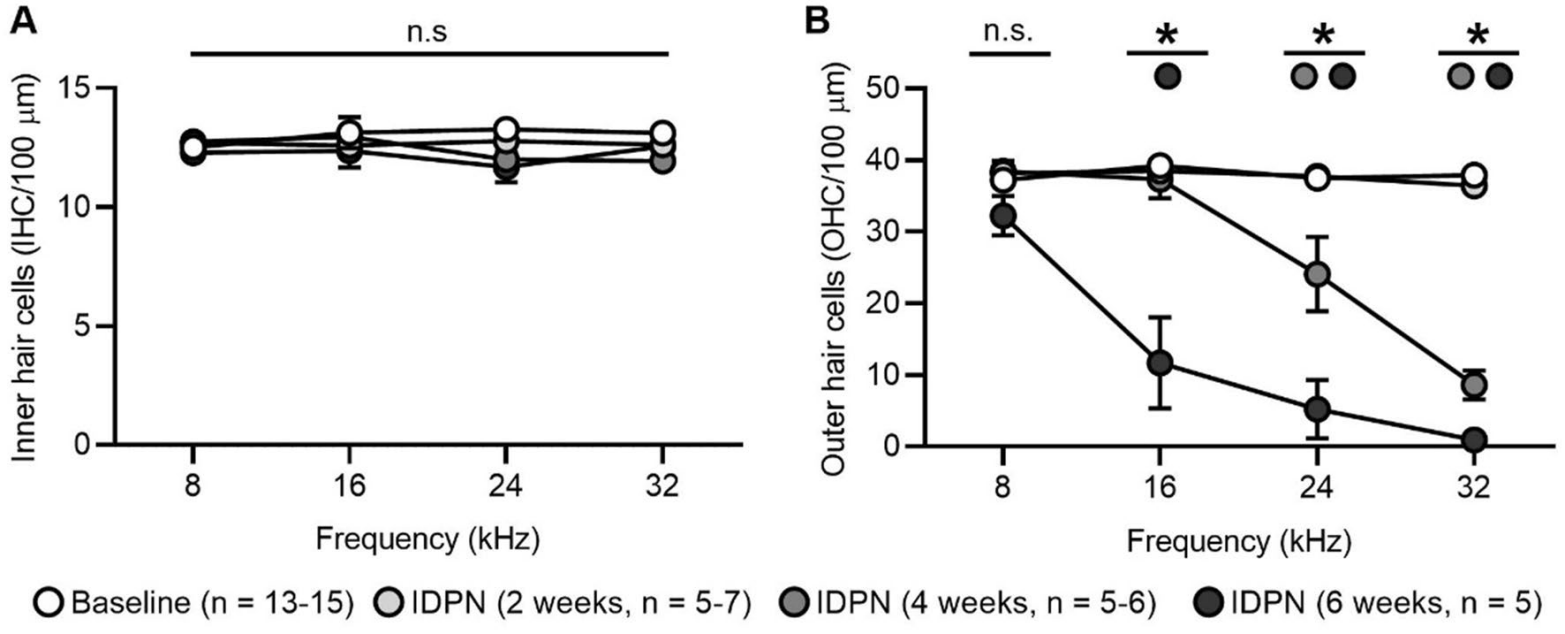
IDPN exposure causes loss of cochlear OHCs. **a**–**b** The numbers of IHCs (**a**) and OHCs (**b**) are shown at four tonotopic regions (corresponding to 8, 16, 24, and 32 kHz) across exposure durations. IHC numbers show no significant (ns) changes across frequency regions in response to IDPN exposure. In contrast, OHC numbers are progressively reduced from high to low frequency regions with increasing IDPN exposure. Asterisks indicate comparisons that are significantly different. Exposure durations include baseline (white circles), 2 weeks (light grey circles), 4 weeks (dark grey circles), and 6 weeks (black circles). The numbers (*n*) of animals assessed are indicated

Both inner and outer hair cell loss was quantified across tonotopic regions (Fig. 4; Table 3). Within a given frequency region, there was no significant difference in the number of IHCs between control mice or mice exposed to IDPN for 2, 4, or 6 weeks (Fig. 4a). In contrast, one-way ANOVA indicated significant differences across time of exposure in OHC counts at the different frequencies [16, 24, 32 kHz; *F*(3,27) = 24.0, *F*(3,23) = 33.1, *F*(3,25) = 227.8, respectively, all *p* ≪ 0.05]. After 4 weeks of IDPN exposure, there was significant loss of OHCs by 36% and 77% in the respective 24 and 32 kHz regions (Fig. 4b). After 6 weeks, there was significant loss of OHCs by 70%, 86%, and 98% in the respective 16, 24, and 32 kHz regions (Fig. 4b). Thus, IDPN exposure causes progressive loss of OHCs from high to low frequency regions of the cochlea, whereas no loss of IHCs was observed in any cochlear region for up to 6 weeks of IDPN exposure.

**Table 3:** Inner and outer hair cell counts following IDPN exposure

Hair cells	Treatment	8 kHz	16 kHz	24 kHz	32 kHz
IHCs (per 100 µm)	Control (2, 4, and 6 weeks, *n* = 13–15)	12.7 ± 0.2 (*n* = 150 IHCs)	12.6 ± 0.2 (*n* = 140 IHCs)	12.8 ± 0.2 (*n* = 130 IHCs)	12.6 ± 0.2 (*n* = 150 IHCs)
	IDPN (2 weeks, *n* = 5–7)	12.8 ± 0.3 (*n* = 60 IHCs)	13.0 ± 0.2 (*n* = 70 IHCs)	12.0 ± 0.1 (*n* = 60 IHCs)	12.0 ± 0.4 (*n* = 50 IHCs)
	IDPN (4 weeks, *n* = 5)	12.5 ± 0.3 (*n* = 60 IHCs)	13.1 ± 0.6 (*n* = 60 IHCs)	13.3 ± 0.4 (*n* = 50 IHCs)	13.1 ± 0.2 (*n* = 50 IHCs)
	IDPN (6 weeks, *n* = 5)	12.3 ± 0.2 (*n* = 50 IHCs)	12.4 ± 0.7 (*n* = 50 IHCs)	11.7 ± 0.6 (*n* = 50 IHCs)	12.6 ± 0.1 (*n* = 50 IHCs)
OHCs (per 100 µm)	Control (2, 4, and 6 weeks, *n* = 12–14)	37.3 ± 1.2 (*n* = 431 OHCs)	39.2 ± 0.6 (*n* = 417 OHCs)	37.3 ± 0.8 (*n* = 384 OHCs)	37.7 ± 0.9 (*n* = 450 OHCs)
	IDPN (2 weeks, *n* = 5–7)	38.1 ± 1.8 (*n* = 177 OHCs)	38.5 ± 1.2 (*n* = 204 OHCs)	37.8 ± 0.5 (*n* = 174 OHCs)	36.4 ± 1.1 (*n* = 146 OHCs)
	IDPN (4 weeks, *n* = 5–6)	38.4 ± 0.7 (*n* = 172 OHCs)	37.3 ± 2.7 (*n* = 166 OHCs)	24.1 ± 5.2 (*n* = 93 OHCs)	8.6 ± 2.0 (*n* = 35 OHCs)
	IDPN (6 weeks, *n* = 5)	32.2 ± 2.7 (*n* = 126 OHCs)	11.7 ± 6.4 (*n* = 47 OHCs)	5.2 ± 4.1 (*n* = 21 OHCs)	0.9 ± 0.9 (*n* = 4 OHCs)

## IDPN exposure causes loss of both cochlear and vestibular afferent synapses

**Fig. 5:**
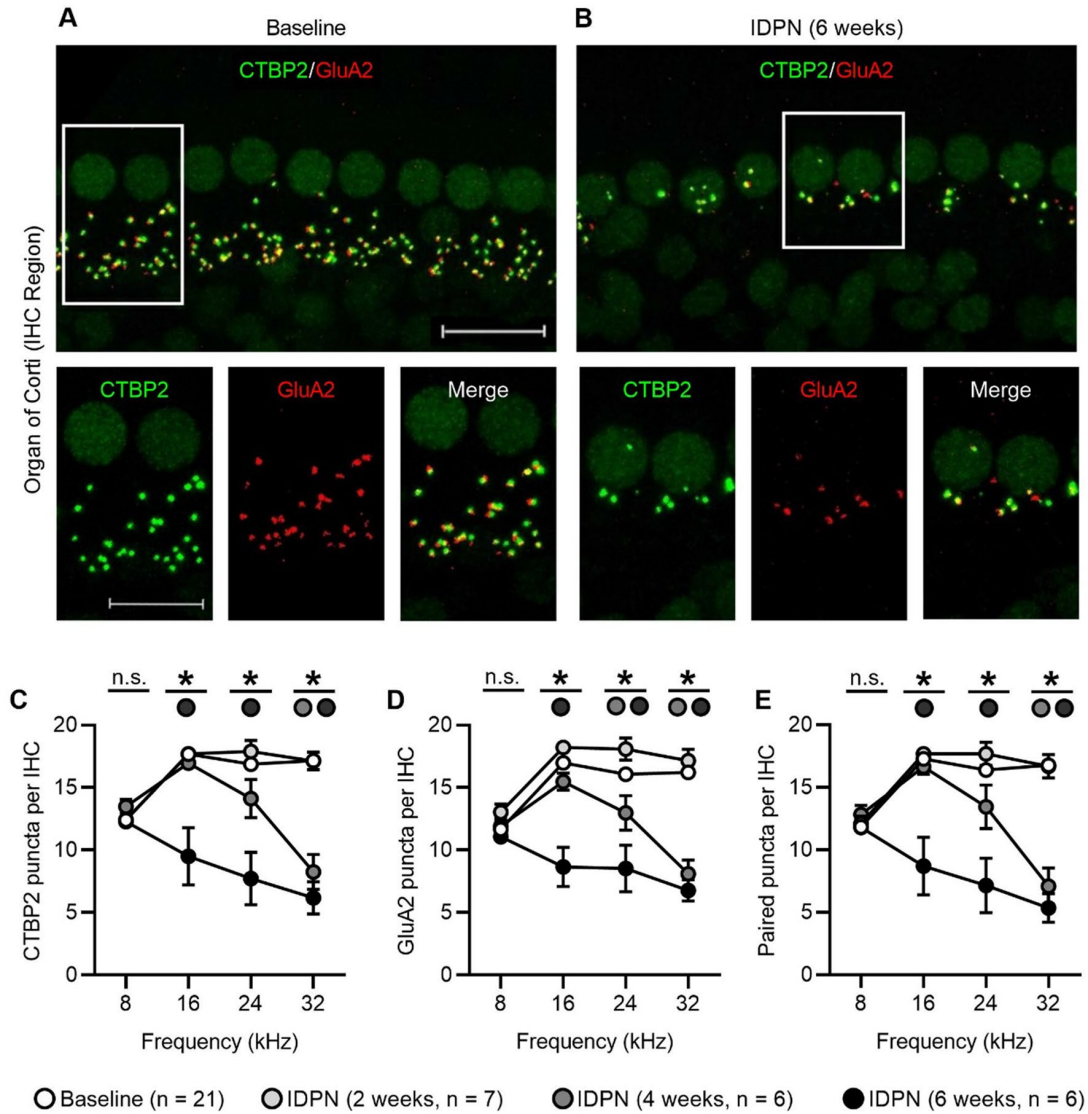
IDPN exposure causes loss of cochlear afferent synapses. **a**, **b** *Z*-projections through the organs of Corti immunostained with anti-CTBP2 to detect IHC nuclei and presynaptic ribbons (green) and anti-GluA2 to detect postsynaptic glutamate receptors (red) are shown at baseline (**a**) and after 6 weeks of IDPN exposure (**b**). Regions of interest are outlined in boxes in the upper panels and shown magnified in the corresponding lower panels. Scale bars equal 15 µm in the upper panels and 10 µm in the lower panels. **c–e** The number of presynaptic elements (CTBP2 puncta per IHC, **c**), postsynaptic elements (GluA2 puncta per IHC, **d**), and paired synaptic elements (paired puncta per IHC, **e**) are quantified at four tonotopic regions (corresponding to 8, 16, 24, and 32 kHz) at the indicated exposure durations (baseline, 2 weeks, 4 weeks, and 6 weeks). Synaptic elements show no significant (ns) changes at the 8 kHz region. Above 8 kHz, however, synaptic elements are progressively reduced from high to low (but greater than 8 kHz) frequency regions with increasing IDPN exposure. In all panels, asterisks indicate comparisons that are significantly different. Exposure durations include baseline (white circles), 2 weeks (light grey circles), 4 weeks (dark grey circles), and 6 weeks (black circles). The numbers (*n*) of animals assessed are indicated (color figure online)

Loss of OHCs (Figs. 3, 4) is consistent with elevated ABR wave I absolute thresholds also observed in response to IDPN exposure (Fig. 1). However, elevated absolute thresholds were observed as early as 2 weeks after IDPN exposure, when no OHC loss was yet detected. For this reason, we suspected that cochlear synaptopathy—loss of synapses between the IHCs and the type I SGN afferent terminals—might also occur in response to IDPN exposure (Fig. 5). To identify synapses, we immunostained organs of Corti isolated from control mice and mice exposed to IDPN after 2, 4, or 6 weeks. Epithelia were immunostained with anti-CTBP2 to detect presynaptic ribbons and anti-GluA2 to detect postsynaptic glutamate receptors, and examined at four different frequency regions (8, 16, 24, and 32 kHz). At baseline (0 weeks, Fig. 5a), CTBP2-labeled presynaptic ribbons were colocalized with GluA2-labeled postsynapses (upper panels and ROIs in lower panels). In contrast, after 6 weeks of IDPN exposure (Fig. 5b), the numbers of CTBP2-labeled presynaptic ribbons and GluA2-labeled postsynapses were reduced (upper panel) and synaptic elements were not always colocalized (lower panels from indicated region of interest).

Synapse loss was quantified across four tonotopic regions for control mice and mice exposed to IDPN for 2, 4, and 6 weeks (Fig. 5c–e; Table 4). Within a given frequency region, there was no significant difference in the number of CTBP2 (Fig. 5c), GluA2 (Fig. 5d), or paired CTBP2-GluA2 (Fig. 5e) puncta per IHC present for controls or after 2 weeks of IDPN exposure. In contrast, after 4 and 6 weeks of IDPN exposure, there was significant loss of CTBP2 [16 kHz: *F*(3,37) = 19.6, 24 kHz: *F*(3,40) = 13.3, 32 kHz: *F*(3,40) = 44.2, all *p* ≪ 0.05], GluA2 [16 kHz: *F*(3,37) = 24.4, 24 kHz: *F*(3,40) = 12.4, 32 kHz: *F*(3,40) = 47.0, all *p* ≪ 0.05], and paired CTBP2-GluA2 [16 kHz: *F*(3,37) = 20.2, 24 kHz: *F*(3,40) = 11.5, 32 kHz: *F*(3,40) = 46.4, all *p* ≪ 0.05] puncta per IHC in all but the lowest frequency region (8 kHz) examined. Specifically, in the 16 kHz region, there was a significant reduction by 50% of paired CTBP2-GluA2 puncta per IHC after 6 weeks of IDPN exposure (*p* ≪ 0.05). In the 24 kHz region, there was a significant reduction by 56% of paired CTBP2-GluA2 puncta per IHC after 6 weeks of IDPN exposure (*p* ≪ 0.05). Finally, in the highest frequency region examined, 32 kHz, there was a significant reduction by 58% and 68% of paired CTBP2-GluA2 puncta per IHC after 4 and 6 weeks, respectively, of IDPN exposure (both *p* ≪ 0.05).

**Table 4:** Synaptic elements per inner hair cell following IDPN exposure

Measure	Treatment	8 kHz	16 kHz	24 kHz	32 kHz
CTBP2/IHC	Control (Baseline, 2, 4, and 6 weeks, *n* = 21)	12.4 ± 0.3 (*n* = 458 IHCs)	17.7 ± 0.4 (*n* = 444 IHCs)	16.9 ± 0.4 (*n* = 411 IHCs)	17.2 ± 0.3 (*n* = 445 IHCs)
	IDPN (2 weeks, *n* = 7–8)	12.4 ± 0.5 (*n* = 108 IHCs)	17.7 ± 0.2 (*n* = 152 IHCs)	17.9 ± 0.9 (*n* = 122 IHCs)	17.1 ± 0.7 (*n* = 110 IHCs)
	IDPN (4 weeks, *n* = 7–10)	13.5 ± 0.6 (*n* = 157 IHCs)	17.0 ± 0.4 (*n* = 176 IHCs)	14.1 ± 1.5 (*n* = 220 IHCs)	8.2 ± 1.4 (*n* = 228 IHCs)
	IDPN (6 weeks, *n* = 5–6)	12.3 ± 0.3 (*n* = 145 IHCs)	9.5 ± 2.3 (*n* = 100 IHCs)	7.7 ± 2.1 (*n* = 131 IHCs)	6.2 ± 1.3 (*n* = 139 IHCs)
GluA2/IHC	Control (Baseline, 2, 4, and 6 weeks, *n* = 21)	11.7 ± 0.3 (*n* = 458 IHCs)	17.0 ± 0.5 (*n* = 444 IHCs)	16.1 ± 0.5 (*n* = 411 IHCs)	16.2 ± 0.4 (*n* = 445 IHCs)
	IDPN (2 weeks, *n* = 7–8)	13.0 ± 0.6 (*n* = 108 IHCs)	18.2 ± 0.2 (*n* = 152 IHCs)	18.1 ± 0.9 (*n* = 122 IHCs)	17.2 ± 0.9 (*n* = 110 IHCs)
	IDPN (4 weeks, *n* = 7–10)	11.9 ± 0.7 (*n* = 157 IHCs)	15.5 ± 0.7 (*n* = 176 IHCs)	13.0 ± 1.4 (*n* = 220 IHCs)	8.1 ± 1.1 (*n* = 228 IHCs)
	IDPN (6 weeks, *n* = 5–6)	11.1 ± 0.4 (*n* = 145 IHCs)	8.6 ± 1.6 (*n* = 100 IHCs)	8.5 ± 1.9 (*n* = 131 IHCs)	6.8 ± 0.9 (*n* = 139 IHCs)
Paired puncta/IHC	Control (Baseline, 2, 4, and 6 weeks, *n* = 21)	11.8 ± 0.4 (*n* = 458 IHCs)	17.3 ± 0.4 (*n* = 444 IHCs)	16.4 ± 0.5 (*n* = 411 IHCs)	16.8 ± 0.3 (*n* = 445 IHCs)
	IDPN (2 weeks, *n* = 7–8)	12.1 ± 0.6 (*n* = 108 IHCs)	17.7 ± 0.2 (*n* = 152 IHCs)	17.7 ± 0.9 (*n* = 122 IHCs)	16.7 ± 0.9 (*n* = 110 IHCs)
	IDPN (4 weeks, *n* = 7–10)	12.8 ± 0.7 (*n* = 157 IHCs)	16.6 ± 0.6 (*n* = 176 IHCs)	13.5 ± 1.8 (*n* = 220 IHCs)	7.1 ± 1.5 (*n* = 228 IHCs)
	IDPN (6 weeks, *n* = 5–6)	11.8 ± 0.4 (*n* = 145 IHCs)	8.7 ± 2.3 (*n* = 100 IHCs)	7.1 ± 2.2 (*n* = 131 IHCs)	5.4 ± 1.2 (*n* = 139 IHCs)

**Fig. 6:**
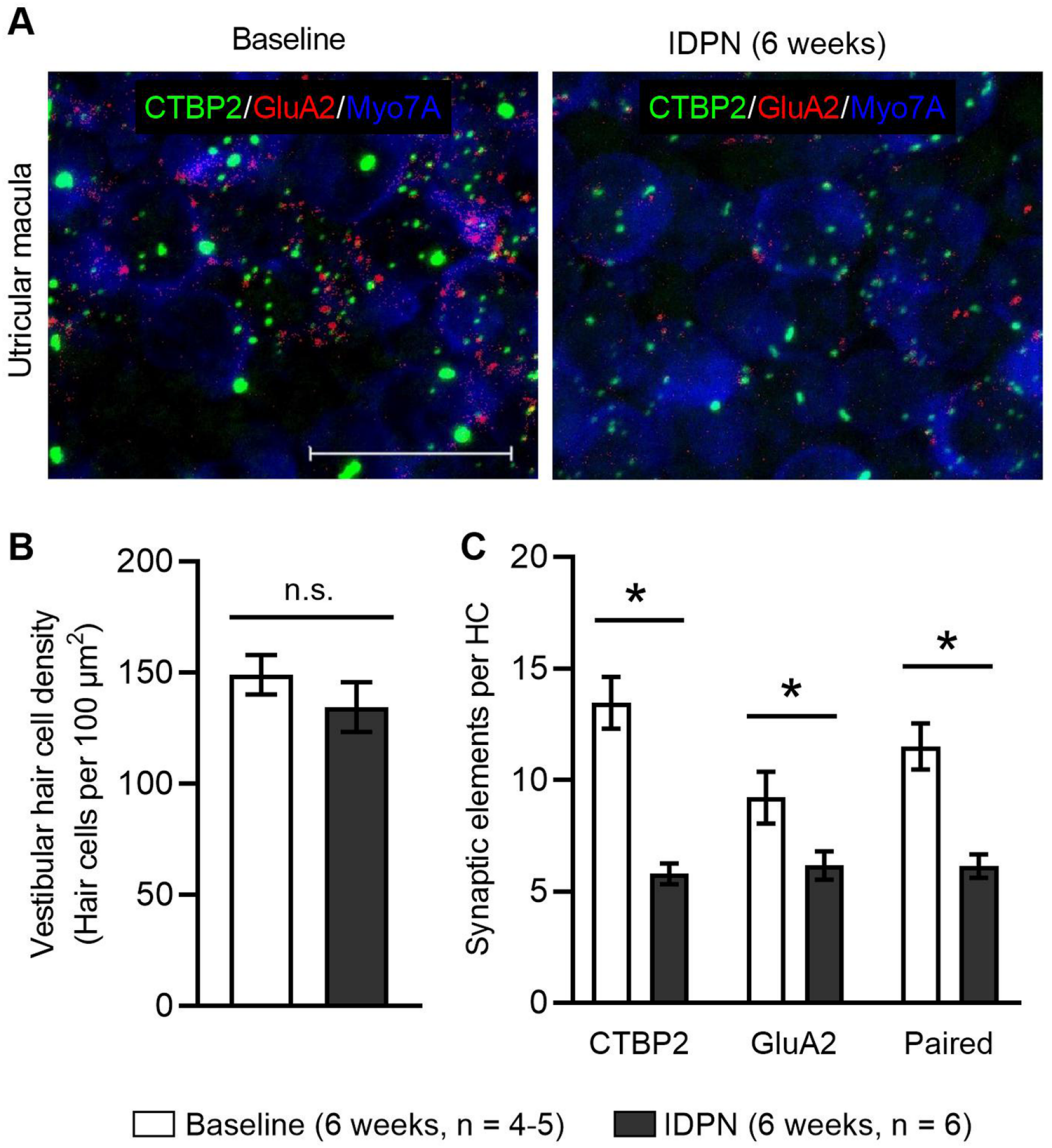
IDPN exposure causes loss of vestibular afferent synapses. **a** Z-projections through the utricular sensory epithelia immunostained with anti-CTBP2 to detect IHC nuclei and presynaptic ribbons (green), anti-GluA2 to detect postsynaptic glutamate receptors (red), and Myo7A to detect HCs (blue) are shown at baseline and after 6 weeks of IDPN exposure. Scale bar equals 15 µm. **b** The number of vestibular HCs is quantified at baseline (white bars) and after 6 weeks of IDPN exposure (black bars). There are no significant (ns) differences. **c** The number of presynaptic elements (CTBP2 puncta per HC), postsynaptic elements (GluA2 puncta per HC), and paired synaptic elements (paired puncta per HC) are quantified at baseline (white bars) and after 6 weeks of IDPN exposure (black bars) and show significant reductions following IDPN exposure (indicated by asterisks) (color figure online)

Loss of vestibular afferent synapses has also been reported in additional vestibular sensory epithelia (cristae) of mice (Greguske et al. 2019) and rats (Sedó-Cabezón et al. 2015) after chronic IDPN exposure. Therefore, we also quantified afferent synapse loss in the vestibular (utricular) sensory epithelium (Fig. 6; Table 5). To identify synapses, we immunostained epithelia from control mice (at 6 weeks) and after 6 weeks of exposure to IDPN (Fig. 6a). To identify synaptic elements per HC, utricular epithelia were immunostained with anti-CTBP2 to detect presynaptic afferent ribbons, anti-GluA2 to detect glutamatergic postsynapses, and anti-MYO7A, a marker of both type I and type II HCs (Hasson et al. 1997). In the striolar region, we found that IDPN exposure had no effect on the numbers of MYO7A-positive HCs (Fig. 6b). In contrast, the number of CTBP2-positive presynaptic ribbons, GluA2-positive postsynapses, and paired synaptic elements per HC were significantly reduced after 6 weeks of IDPN exposure (unpaired *t* test, *p* ≪ 0.05, *p* = 0.036, *p* = 0.001, respectively, Fig. 6c).

**Table 5:** Synaptic elements per vestibular hair cell following IDPN exposure

Measure	Treatment	Striolar region
CTBP2/HC	Control (6 weeks, *n* = 5)	13.5 ± 1.2 (*n* = 745 HCs)
	IDPN (6 weeks, *n* = 6)	5.8 ± 0.5 (*n* = 807 HCs)
GluA2/HC	Control (6 weeks, *n* = 4)	9.2 ± 1.2 (*n* = 745 HCs)
	IDPN (6 weeks, *n* = 6)	6.2 ± 0.6 (*n* = 807 HCs)
Paired puncta/HC	Control (6 weeks, *n* = 4)	11.5 ± 1.0 (*n* = 745 HCs)
	IDPN (6 weeks, *n* = 6)	6.1 ± 0.5 (*n* = 807 HCs)

## IDPN exposure causes loss of CASPR1 from the calyx-shaped afferent terminals of the vestibular neurons, but not from the paranodal region of the type I SGN afferent terminals

After 2 weeks of IDPN exposure, mice showed elevated ABR wave I absolute thresholds, but examination of cochlear afferent synapses revealed no synaptopathy (Fig. 5) or outer hair cell loss (Fig. 3). Thus, we further examined the molecular architecture of the type I SGN afferent terminals to identify the pathology contributing to early hearing loss after IDPN exposure. In the vestibular sensory epithelia, CASPR1 is enriched in the calyx-shaped afferent terminals contacting the type I HCs (Sousa et al. 2009). Previous work has shown that dismantlement of the calyceal junction, as evidenced by loss of CASPR1 immunoreactivity, is an early event in the afferent damage induced by IDPN exposure (Greguske et al. 2019; Sedo-Cabezón et al. 2015). In the type I SGN afferent terminals of the cochlea, CASPR1 is found at the first heminodes and subsequent paranodes (Hossain et al. 2005), where it anchors and organizes ion channels essential for action potential generation.

**Fig. 7:**
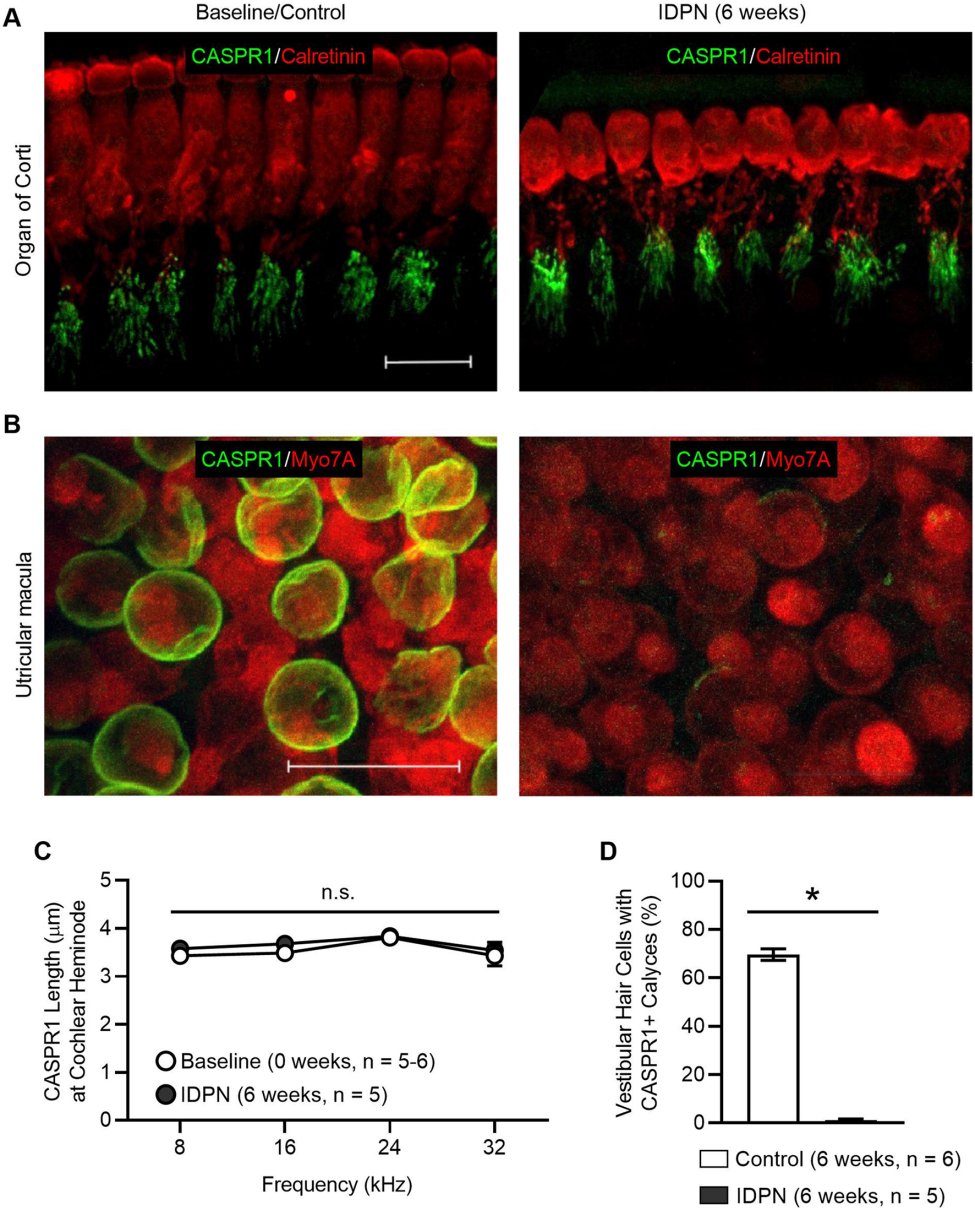
IDPN exposure causes loss of CASPR1 from the calyx-shaped afferent terminals of the vestibular neurons but not from the paranodal region of the type I SGN afferent terminals. **a** *Z*-projections through the organs of Corti immunostained with anti-CASPR1 (green) and anti-calretinin to detect IHCs (red) are shown at baseline (left panel) and after 6 weeks of IDPN exposure (right panel). Scale bar equals 15 µm. **b** *Z*-projections through the utricular sensory epithelia immunostained with anti-CASPR1 (green) and anti-Myo7A to detect HCs (red) are shown at baseline (left panel) and after 6 weeks of IDPN exposure (right panel). Scale bar equals 15 µm. **c** The distribution of CASPR1 in the cochlear heminodes (length) is quantified at four tonotopic regions (corresponding to 8, 16, 24, and 32 kHz) at baseline (white circles) and after 6 weeks of IDPN exposure (black circles). There were no significant (ns) differences in CASPR1 distrbution. **d** The number of CASPR1-positive calyces is quantified in control (white bars) and IDPN-exposed (black bars) animals at 6 weeks and show significant reductions following IDPN exposure (indicated by asterisks) (color figure online)

To investigate whether IDPN exposure causes a similar loss of CASPR1 in type I SGN afferent terminals, we immunostained organs of Corti isolated from control mice (6 weeks) and after 6 weeks of exposure to IDPN with anti-calretinin to label IHCs and the type I SGN afferent terminals and anti-CASPR1 (Fig. 7a). No qualitative difference in CASPR1 immunoreactivity was seen following IDPN exposure for either 4 (data not shown) or 6 weeks in comparison to control mice (Fig. 7a). To examine the distribution of CASPR1 more carefully, we quantified the length of CASPR1 distribution in at least 6 afferent terminals per IHC and at least 6 IHCs per animal across 4 frequency regions (Fig. 7c; Table 6). No significant differences (unpaired t tests) in distribution were observed.

**Table 6:** Quantification of CASPR1 expression in the organ of Corti following IDPN exposure

Measure	Treatment	8 kHz	16 kHz	24 kHz	32 kHz
CASPR1 Length (µm) in cochlear type I afferents	Control (6 weeks, *n* = 5–6)	3.4 ± 0.1	3.5 ± 0.1	3.8 ± 0.1	3.4 ± 0.2
	IDPN (6 weeks, *n* = 5)	3.6 ± 0.04	3.7 ± 0.1	3.8 ± 0.1	3.5 ± 0.2

In contrast to our findings in the organs of Corti, but consistent with previous observations (Greguske et al. 2019; Sedó-Cabezón et al. 2015), parallel examination revealed complete loss of CASPR1 immunoreactivity in the striola of the utricular macula in response to IDPN exposure (Fig. 7b). In these experiments, vestibular sensory epithelia were isolated from control mice (at 6 weeks) and after 6 weeks of exposure to IDPN and immunostained with anti-MYO7A to label vestibular hair cells and anti-CASPR1 (Fig. 7b). Quantification revealed no loss of MYO7A-positive HCs in response to IDPN exposure (see Fig. 6b), but a dramatic reduction in the percentage of vestibular HCs associated with CASPR1-positive calyces after 6 weeks of IDPN exposure (1.1 ± 0.6%) compared to control (69.6 ± 2.4%, Fig. 7d) was observed (unpaired *t* test, *p* ≪ 0.05). These experiments show that loss of CASPR1 in response to IDPN exposure is specific to the calyceal junctions of the vestibular sensory epithelia and not observed in the paranodal regions of the type I SGN afferent dendrites in the organ of Corti.

## Auditory function recovers following cessation of short-duration IDPN exposure

**Fig. 8:**
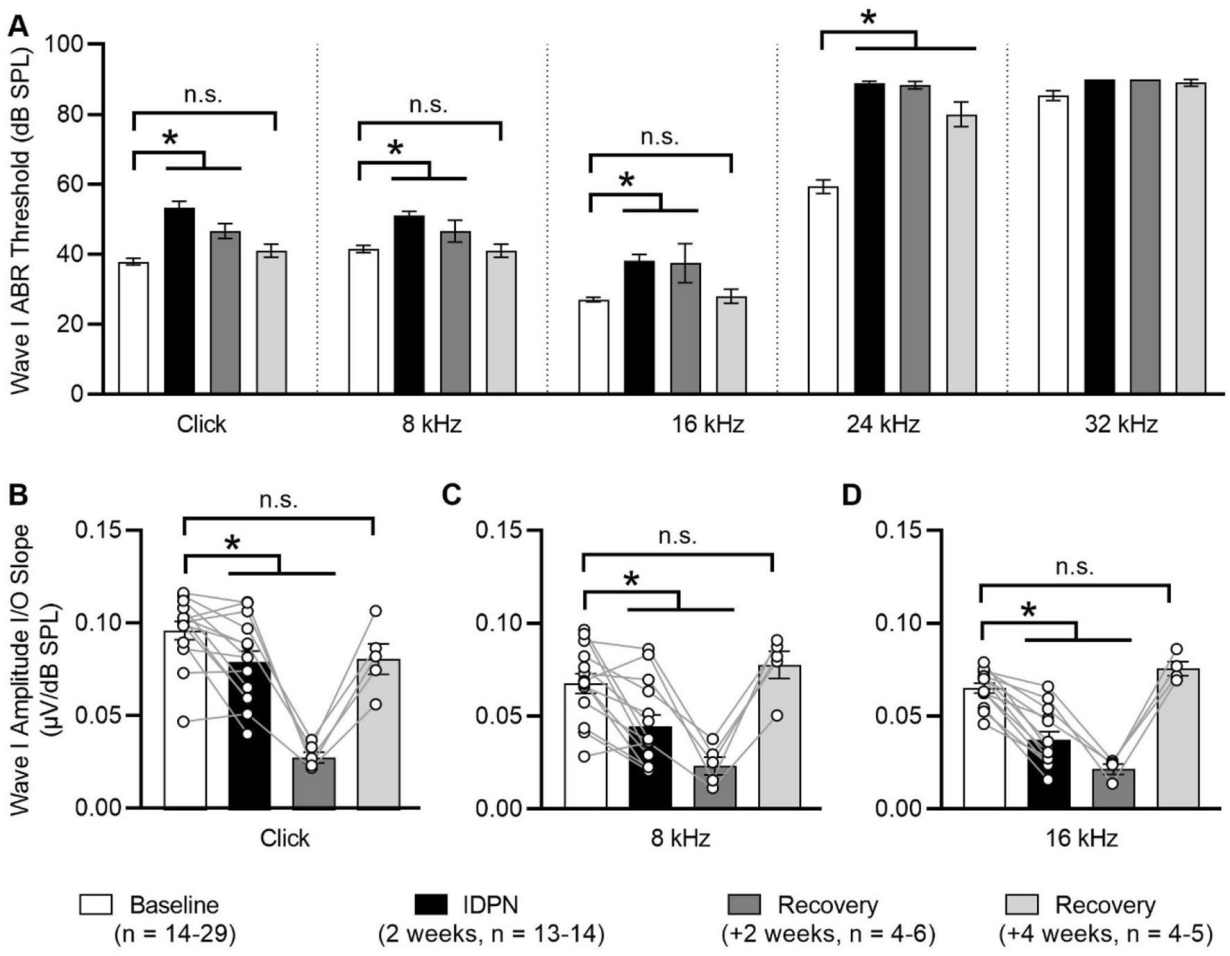
IDPN exposure followed by recovery causes hearing loss followed by recovery assessed by ABR measurements. **a**–**c** Mean absolute ABR wave I thresholds (**a**) and wave I amplitude I/O slopes (**b**–**d**) are shown across exposure/recovery durations for click stimuli and the indicated pure tone frequencies. Wave I thresholds increase with IDPN exposure and then recover to pre-exposure values after 4 weeks of recovery. Wave I amplitudes decrease with IDPN exposure and recover to pre-exposure values after 4 weeks of recovery. In all panels, asterisks indicate significant differences and ns indicates non-significant differences. Exposure/recovery durations are indicated as baseline (white bars), 2 weeks of IDPN exposure (black bars), 2 weeks of recovery (dark grey bars), and 4 weeks of recovery (light grey bars). The numbers (*n*) of animals assessed are indicated

The lack of observable structural pathology after 2 weeks of IDPN exposure led us to speculate that elevated ABR wave I absolute thresholds observed after 2 weeks of IDPN exposure may recover to baseline values following cessation of IDPN exposure. To test this hypothesis, we exposed mice to IDPN for 2 weeks and then allowed either 2 or 4 weeks of recovery (Fig. 8; Table 7). In comparison to baseline values (0 weeks), wave I ABR thresholds were significantly elevated for click stimuli and pure tones (8, 16, and 24 kHz) after 2 weeks of IDPN exposure (Fig. 8a). Wave I ABR amplitude I/O slopes were also decreased for click stimuli (Fig. 8b) and for pure tones of 8 (Fig. 8c) and 16 kHz (Fig. 8d). Data from baseline (0 weeks) and IDPN (2 weeks) are re-plotted from Fig. 1 to only include the animals tracked in the recovery experiments. Because ABR wave I amplitude responses were greater than 80 dB SPL when measured in response to 24 and 32 kHz pure tones after 2 weeks of IDPN exposure, ABR wave I amplitude I/O slopes were not calculated for these frequencies. Because ABR wave I latency I/O slopes did not show significant differences after 2 weeks of exposure (Fig. 1c) these values were not calculated for the recovery experiments.

**Table 7:** Wave I ABR absolute thresholds and wave I amplitude I/O slopes following IDPN exposure and subsequent recovery

Measure	Treatment	Click	8 kHz	16 kHz	24 kHz	32 kHz
Wave I absolute thresholds (dB SPL)	Baseline (*n* = 29)	37.9 ± 1.0	41.6 ± 1.0	27.1 ± 0.6	59.3 ± 1.9	85.3 ± 1.4
	IDPN (2 weeks, *n* = 14)	53.2 ± 1.9	51.1 ± 1.2	38.2 ± 1.8	88.9 ± 0.6	> 90
	Recovery (+ 2 weeks, *n* = 6)	46.7 ± 2.1	46.7 ± 3.1	37.5 ± 5.6	88.3 ± 1.1	> 90
	Recovery (+ 4 weeks, *n* = 5)	41.0 ± 1.9	41.0 ± 1.9	28.0 ± 2.0	80.0 ± 3.5	89.0 ± 1.0
Wave I amplitude I/O slopes (µV/dB SPL)	Baseline (*n* = 13–14)	0.097 ± 0.005 (*n* = 14)	0.068 ± 0.005 (*n* = 14)	0.066 ± 0.003 (*n* = 13)	–	–
	IDPN (2 weeks, *n* = 13–14)	0.080 ± 0.006 (*n* = 14)	0.045 ± 0.006 (*n* = 14)	0.038 ± 0.004 (*n* = 13)	–	–
	Recovery (+ 2 weeks, *n* = 4–5)	0.028 ± 0.003 (*n* = 5)	0.024 ± 0.005 (*n* = 5)	0.022 ± 0.003 (*n* = 4)	–	–
	Recovery (+ 4 weeks, *n* = 4–5)	0.081 ± 0.008 (*n* = 5)	0.078 ± 0.007 (*n* = 5)	0.076 ± 0.004 (*n* = 4)	–	–

After 4 weeks of recovery from IDPN exposure, ABR wave I absolute thresholds for click stimuli and 8 and 16 kHz pure tones recovered to baseline values [*F*(3,50) = 22.5, *F*(3,50) = 11.1, *F*(3,50) = 12.8, respectively, all *p* ≪ 0.05], but not for 24 kHz pure tones. ABR wave I amplitude I/O slopes were also not significantly different between values measured at baseline and after 4 weeks of recovery (Fig. 8b–d). After 2 weeks of IDPN intoxication, ABR wave I amplitude I/O slopes were significantly reduced for the click stimuli and the 8 and 16 kHz pure tones compared to baseline [*F*(1.438,10.07) = 25.4, *p* = 0.02, *F*(1.337,9.359) = 18.8, *p* = 0.008, *F*(1.613,9.678) = 54.7, *p* ≪ 0.05, respectively, Fig. 8b–d]. Surprisingly, after 2 weeks of recovery, ABR wave I absolute thresholds were not fully recovered and, in fact, ABR wave I amplitude I/O slopes remained significantly reduced in comparison to baseline values for click stimuli and 8 and 16 kHz pure tones (*p* = 0.003, *p* = 0.005, *p* = 0.005, respectively, Fig. 8b–d).Together, these data show that after cessation of IDPN exposure—at least at exposure durations that are not associated with structural pathology—wave I ABR responses continue to worsen but ultimately recover to baseline values.

## Discussion

In this study, we characterized the effects of chronic administration of IDPN (at doses known to cause peripheral vestibular ototoxicity) on the cochlea, the auditory end organ. Cochlear pathology was quantified using ABR measurements and immunohistological assessments of the IHCs and OHCs and the synapses between the IHCs and type I SGNs. Functionally, chronic IDPN exposure caused auditory thresholds that progressively worsened from high to low frequencies. Morphologically, chronic IDPN exposure caused outer but not inner HC loss that also progressed from high to low frequency regions of the cochlea. The number of synapses between the IHCs and type I SGNs was also reduced at higher frequencies with chronic IDPN exposure. With continued exposure, structural pathology manifested as parallel loss of OHCs and loss of synapses between the IHCs and type I SGNs. Importantly, auditory thresholds were reduced before evidence of structural pathology, and auditory thresholds recovered fully if IDPN administration was stopped before evidence of structural pathology.

These findings are consistent with previous characterization of the cochlear pathology in response to IDPN exposure in rats and guinea pigs. Previous work in rats exposed sub-acutely to IDPN showed that dysfunction is dose-dependent, occurs first in high frequency regions of the cochlea (examined using reflex modification audiometry: Crofton and Knight 1991), and, with sufficiently high doses, occurs in both high and low frequency regions of the cochlea (examined using ABR to filtered click stimuli: Crofton et al. 1994). Moreover, and consistent with our findings, examination of suprathreshold ABRs revealed decreased wave I amplitudes but no statistically significant differences in wave I latencies (Crofton et al. 1994). In contrast to our findings, previous examination of IDPN-exposed rats revealed that the highest dosages were associated with pathology throughout the cochlea, including not only the organ of Corti but also the stria vascularis, the SGNs, and the cochlear nerve (Crofton et al. 1994). We did not examine the morphology of the stria vascularis, the SGNs, or the cochlear nerve in these experiments but suspect that pathology of these structures would only be apparent at extended doses under our administration regime.

Importantly, our work is unique in identifying the earliest stages of cochlear pathology in response to chronic IDPN exposure and, thereby, establish a model to identify the mechanisms responsible for the earliest and likely most reversible stages of hearing loss during chronic ototoxicity. First, we specifically examined the integrity of the afferent synapses between the IHCs and type I SGN afferent dendrites. These synapses have received increasing attention as the cochlear structures most vulnerable to noise-induced and age-related loss (Liberman and Kujawa 2017). In our experiments, loss of afferent synapses and OHCs in response to IDPN exposure occurred with a similar spatial (tonotopic) and temporal progression and was associated with functional loss, as revealed by ABR data, in absence of significant IHC loss. A parallel loss of OHCs and type I SGN afferent terminals largely exceeding IHC loss has also been previously reported in an acute gentamicin model, but these authors did not examine auditory function and did not associate the pathological changes to frequency regions of the cochlea (Ruan et al. 2014). While the greater susceptibility of OHCs compared to IHCs to ototoxic insults is well established, the co-occurrence of cochlear synaptopathy has not been previously identified. Our findings together with previous findings from mice (Ruan et al. 2014) and humans (Hinojosa and Lerner 1987; Sone et al. 1998) support the conclusion that cochlear synaptopathy has a significant role in ototoxic-induced hearing loss. Additional work is necessary to establish whether the auditory synapses are more vulnerable than OHCs to exposure to IDPN and other ototoxic compounds, particularly in chronic exposure paradigms. As discussed below, the recovery data in the present work support this prediction.

In this work, we also compared the physiological, behavioral, and morphological pathology between the auditory and vestibular end organs in IDPN-exposed mice. There were notable similarities and differences in the functional, morphological, and molecular pathology in response to IDPN exposure between these two structures. Functionally, auditory dysfunction assessed by ABR was correlated to vestibular dysfunction assessed behaviorally; however, auditory dysfunction was detected before vestibular dysfunction could be detected and also progressed more rapidly. These findings suggest that sensorineural structures in the cochlea may be more vulnerable to IDPN exposure in comparison to sensorineural structures in the vestibule. This difference in vulnerability may be influenced by differences in the innate regenerative capacity of these structures. Even in response to persistent end organ damage, vestibular function shows some recovery (Cassel et al. 2019), and regeneration of HCs following IDPN exposure has been specifically reported (Sayyid et al. 2019). In contrast, equivalent regeneration and recovery of sensorineural structures has not been reported in the cochlea. Thus, the reduced regenerative capacity of the cochlea in comparison to the vestibule may contribute to the greater vulnerability of the cochlea in comparison to the vestibule in response to IDPN exposure. Further work is necessary to substantiate this hypothesis.

Morphologically, in both the cochlea and vestibule, we observed loss of afferent synapses. However, we observed extensive loss of cochlear HCs, specifically OHCs, but no loss of vestibular HCs. Vestibular HC loss has been reported previously in mice and other species using different IDPN administration schemes, including chronic drinking water exposure (Greguske et al. 2019; Llorens et al. 1993; Llorens and Rodriguez-Farre 1997; Soler-Martin et al. 2007). Thus, vestibular HC loss would have been observed had IDPN been administered at higher doses and/or longer durations in these experiments. Nevertheless, sparing of vestibular but not cochlear HCs supports the hypothesis of increased cochlear sensitivity to IDPN exposure, at least in this species and administration regime.

Molecularly, we observed no change in the expression or distribution of CASPR1, a key component of mammalian paranodal junctions (Einheber et al. 1997), in type I SGN afferent terminals in the cochlea in response to IDPN exposure. In contrast, this and previous (Greguske et al. 2019; Sedó-Cabezón et al. 2015) work showed complete loss of CASPR1 in the calyx-shaped afferent terminals contacting the type I HCs in the vestibule in response to IDPN exposure. Even under the IDPN administration regime used in this study, where no loss of vestibular HCs occurred, CASPR1 immunoreactivity was nevertheless completely abolished. We suspect the different pathologies reflect different functions served by CASPR1 in these two structures: organization of action potential initiation zones in the type I SGN afferent terminals in the cochlea versus synaptic adhesion in the calyceal junctions in the vestibule. In support of this speculation, paranodal CASPR1 is maintained in the vestibular afferent axons despite loss of CASPR1 in the calyceal junctions of the same afferents in response to IDPN exposure (unpublished findings).

By characterizing the earliest stages of IDPN-induced chronic cochlear toxicity, we found that cochlear dysfunction evidenced by elevated auditory thresholds was detected before morphological (i.e., loss of outer hair cells and auditory synapses) or molecular pathology (i.e. loss of CASPR1 immunoreactivity) was observed. Although we cannot rule out the possibility of undetected deficits, such as demyelination and/or disorganization of the spike initiation zone (e.g., Wan and Corfas 2017) or alterations in medial or lateral efferent innervation (Ruan et al. 2014), our observation that ABR wave I latencies were unchanged by IDPN exposure argues against these possibilities.

While this observation does not per se suggest that the auditory synapses are more vulnerable than OHCs to IDPN exposure, experiments examining the recovery of auditory thresholds and suprathreshold responses following cessation of IDPN exposure suggest greater vulnerability of the synapses. Specifically, following cessation of IDPN administration, ABR wave I absolute thresholds showed recovery (albeit incomplete in tonotopic regions at and above 24 kHz), whereas ABR wave I suprathreshold amplitudes continued to worsen (decrease). Ultimately, suprathreshold amplitudes recovered to their baseline (preexposure) values, and the recuperation of ABR wave I absolute thresholds shows almost a complete recovery as well. Normal ABR wave I absolute thresholds with reduced suprathreshold amplitudes, for example following noise exposure, has been interpreted as evidence of cochlear synaptopathy—hearing impairment resulting from disrupted function and/or loss of the synapses between the IHCs and type I SGNs (Liberman and Kujawa 2017; Moser and Starr 2016). Thus, our observation of worsening suprathreshold ABR wave I amplitudes despite improving ABR wave I absolute thresholds is consistent with functional pathology of the synapses. Functional pathology of the OHCs, on the other hand, would not be expected to yield this phenotype. Our observations of hearing impairments consistent with cochlear synaptopathy following IDPN exposure suggest that synaptic uncoupling may be an early phase of cochlear synaptopathy. Synaptic uncoupling may be more difficult to discern in the bouton-shaped afferent terminals in the cochlea compared to the calyx-shaped afferent terminals in the vestibule.

Ultimately, we found that auditory function recovered fully if IDPN administration was stopped before evidence of morphological pathology, including loss of outer hair cells and auditory synapses. Importantly, we cannot exclude the possibility that more subtle molecular pathology, such as ion channel or myelin reorganization, went unobserved in our experiments and contributed to IDPN-induced pathophysiology. Nevertheless, we suspect that disruption of adhesion complexes between the IHCs and type I SGN afferent terminals (synaptic uncoupling) may be the earliest and still reversible stage of IDPN-induced ototoxicity. We further suspect that functional recovery is no longer possible after synaptic uncoupling has progressed to morphological pathology evidenced by loss of synapses. Future work is necessary to substantiate these speculations and should additionally examine if there are differences in vulnerability among subgroups of afferent synapses (as has been reported in response to noise-induced synaptopathy: Liberman and Kujawa 2017). Finally, additional work should examine IDPN-induced pathology of the efferent synapses contacting the OHCs, especially in comparison to the time course of OHC loss. These efferent synapses are known to be susceptible to gentamicin-induced damage independently of OHC loss (Ruan et al. 2014).

Moreover, our comparison of pathology between end organs of the inner ear indicated greater vulnerability of the cochlea than vestibule in response to IDPN exposure. This finding would be better corroborated using equivalent measures of auditory and vestibular function. Thus, future work should additionally investigate vestibular function using measurements of vestibular evoked potentials (e.g. Braude et al. 2015). Indeed, earlier work comparing ABRs and VsEPs showed strain-dependent differences in the vulnerability to age-related loss of auditory and vestibular function in mouse (Jones et al. 2006). Genetic factors likely contribute to differences in the relative severity and rate of loss of function between the auditory and vestibular end organs. Thus, our work establishes a model in which the molecular mechanisms that alter the intrinsic vulnerability to ototoxicity can be identified and manipulations targeting these mechanisms to protect against inner ear damage can be tested.

In conclusion, this work identifies previously unrecognized forms of IDPN-induced damage in the cochlea that results in synaptopathy, likely due to uncoupling of the IHCs and type I SGN afferent terminals. Importantly, the earliest stages of IDPN-induced synaptopathy were reversible. Future work is needed to identify the underlying molecular mechanisms, especially of these earliest and reversible stages. Identification of these mechanisms would guide development of novel therapeutic strategies to protect against hearing loss in response to ototoxic agents and other forms of acquired hearing loss.

## Supplementary Information

Below is the link to the electronic supplementary material.

Supplementary file1 (DOCX 329 KB)

## Data Availability

The datasets generated during and/or analyzed during the current study are available from the corresponding author on reasonable request.
