# Supplementary material for: Assessment of cochlear toxicity in response to chronic 3,3′-iminodipropionitrile in mice reveals early and reversible functional loss that precedes overt histopathology
Source: Arch Toxicol. 2021 Jan 25;95(3):1003–21. doi: 10.1007/s00204-020-02962-5 (PMC7904549; doi:10.1007/s00204-020-02962-5)
Supplement: Supplementary file 1 — Supplementary file1 (DOCX 329 KB) [file 204_2020_2962_MOESM1_ESM.docx]

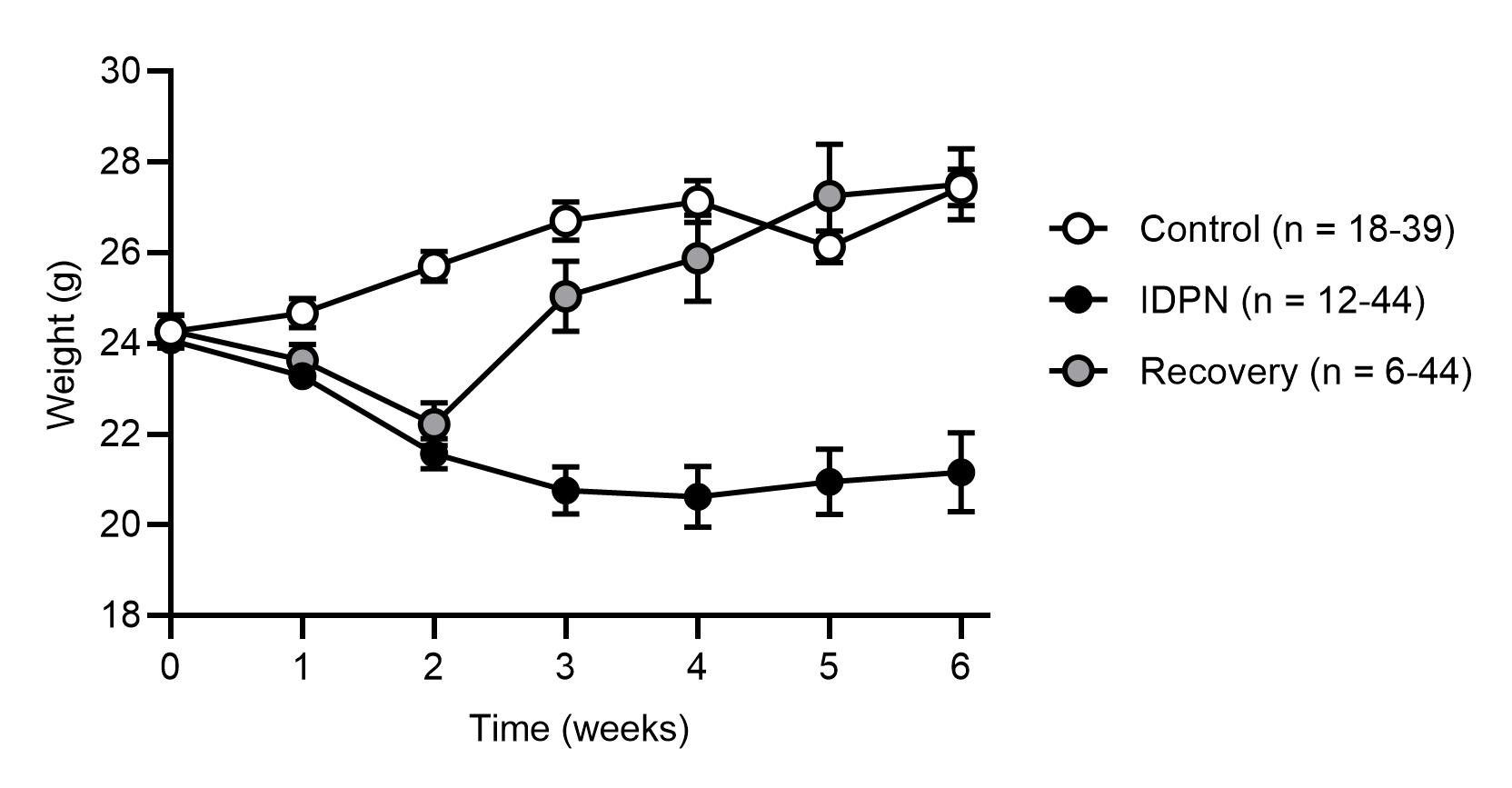


**Supplemental Figure 1. IDPN exposure causes changes in weight.** Control mice showed steady weight gain over the experimental time course (white circles). IDPN exposure caused progressive weight loss that stabilized at approximately 3 weeks (black circles). IDPN exposure for 2 weeks followed by recovery caused progressive weight loss that recovered following cessation of IDPN administration (grey circles).


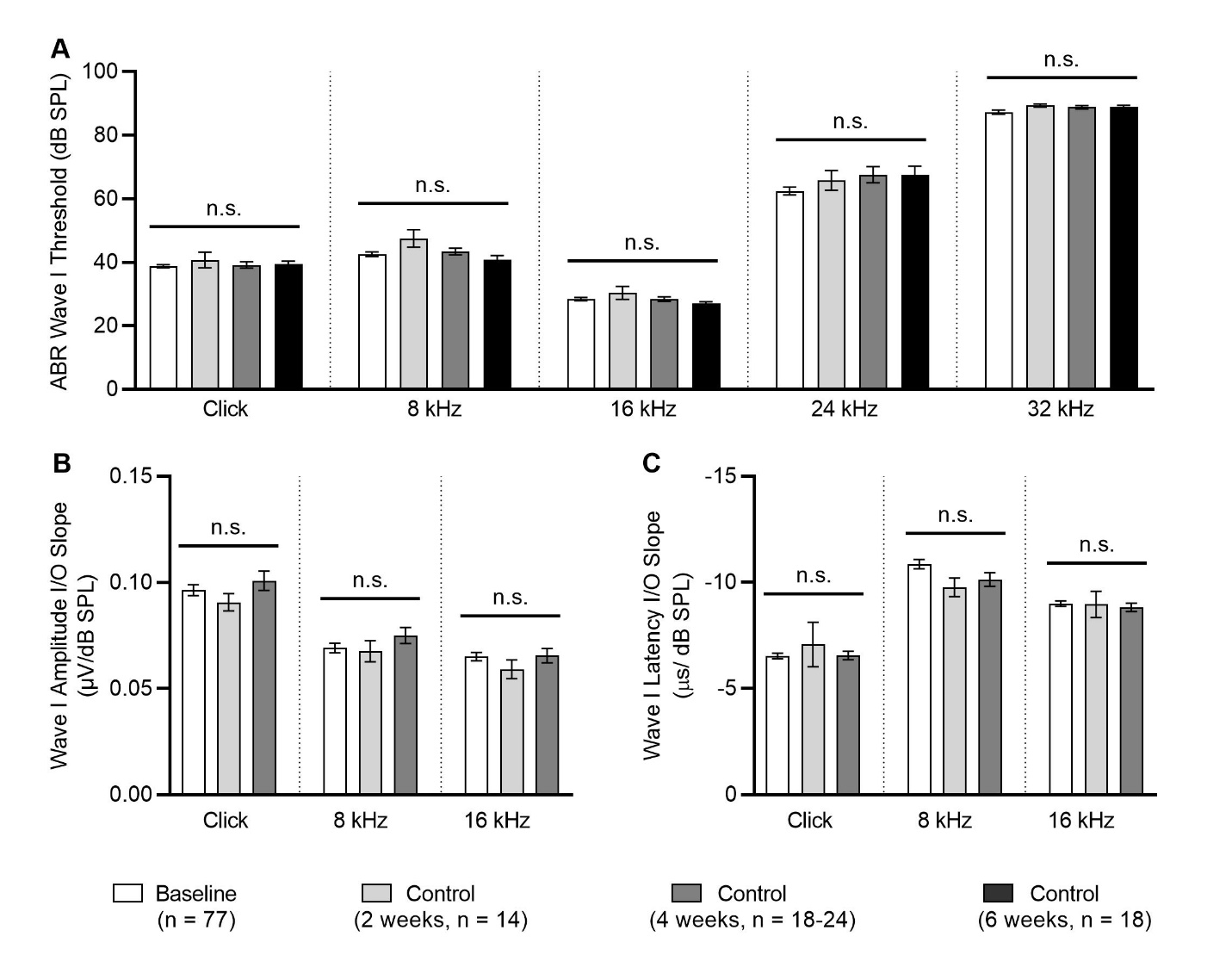


**Supplemental Figure 2. Control animals show no changes in auditory function assessed by ABR measurements. A-C.** Mean absolute ABR wave I thresholds (**A**), wave I amplitude I/O slopes (**B**), and wave I latency I/O slopes (**C**) are shown across exposure durations for the indicated click stimuli and pure tone frequencies. Wave I thresholds, wave I amplitudes, and wave I latencies show no significant (n. s.) changes across frequencies or control exposure durations. In all panels, durations are indicated as baseline (white bars), 2 weeks (light grey bars), 4 weeks (dark grey bars), and 6 weeks (black bars). The numbers (n) of animals assessed are indicated.

**Supplemental Table 1. Body Weight during IDPN Exposure**

|  | Weight (g) | | | | | | |
| --- | --- | --- | --- | --- | --- | --- | --- |
| Treatment | Week 0 | Week 1 | Week 2 | Week 3 | Week 4 | Week 5 | Week 6 |
| Control | 24.3±0.4  (n=39) | 24.7±0.3  (n=32) | 25.7±0.3  (n=32) | 26.7±0.4  (n=24) | 27.1±0.5  (n=24) | 26.1±0.4  (n=18) | 27.5±0.4  (n=18) |
| IDPN | 24.1±0.2  (n=44) | 23.3±0.3  (n=38) | 21.6±0.3  (n=38) | 20.8±0.5  (n=24) | 20.6±0.7  (n=24) | 21.0±0.7  (n=12) | 21.2±0.9  (n=12) |
| Recovery | 24.3±0.4  (n=14) | 23.6±0.4  (n=14) | 22.2±0.5  (n=14) | 25.1±0.8  (n=6) | 25.9±1.0  (n=6) | 27.3±1.1  (n=6) | 27.5±0.8  (n=6) |

**Supplemental Table 2. Wave I ABR absolute thresholds and wave I amplitude and latency I/O slopes (Control)**

| **Measure** | **Treatment** | **Click** | **8 kHz** | **16 kHz** | **24 kHz** | **32 kHz** |
| --- | --- | --- | --- | --- | --- | --- |
| **Wave I absolute thresholds (dB SPL)** | Baseline  (n = 77) | 38.7 ± 0.5 | 42.5 ± 0.7 | 28.4 ± 0.5 | 62.4 ± 1.2 | 87.2 ± 0.7 |
|  | Control  (2 weeks,  n = 14) | 40.7 ± 2.5 | 47.5 ± 2.8 | 30.4 ± 2.1 | 65.7 ± 3.1 | 89.3 ± 0.5 |
|  | Control  (4 weeks,  n = 24) | 39.2 ± 1.0 | 43.3 ± 1.1 | 28.3 ± 0.7 | 67.5 ± 2.5 | 88.8 ± 0.5 |
|  | Control  (6 weeks,  n = 18) | 39.4 ± 0.9 | 40.8 ± 1.3 | 26.9 ± 0.6 | 67.5 ± 2.7 | 88.9 ± 0.5 |
| **Wave I Amplitude I/O slopes (µV/dB SPL)** | Baseline  (n = 77) | 0.096 ± 0.003 | 0.069 ± 0.002 | 0.065 ± 0.002 | - | - |
|  | Control  (2 weeks,  n = 14) | 0.091 ± 0.004 | 0.068 ± 0.005 | 0.059 ± 0.004 | - | - |
|  | Control  (4 weeks,  n = 18) | 0.101 ± 0.005 | 0.075 ± 0.004 | 0.065 ± 0.003 | - | - |
| **Wave I Latency I/O slopes (µs/dB SPL)** | Control  (n = 77) | -6.53 ± 0.14 | -10.9 ± 0.22 | -9.00 ± 0.13 | - | - |
|  | Control  (2 weeks,  n = 14) | -7.08 ± 1.05 | -9.77 ± 0.44 | -8.97 ± 0.62 | - | - |
|  | Control  (4 weeks,  n = 18) | -6.55 ± 0.20 | -10.13 ± 0.33 | -8.83 ± 0.20 | - | - |
